# Serotonergic System‐Targeted Nucleic Acid Hydrogel Coordinates Excitability Restoration and Circuit Reconstruction for Spinal Cord Injury Therapy

**DOI:** 10.1002/adma.202521427

**Published:** 2026-01-13

**Authors:** Chunlin Li, Xiaoqing Zhao, Kai Jiang, Haixia Kang, Shuo Liu, Linlin Jiang, Baoshuai Bai, Haonan Cui, Ziyang Zhang, Menglei Dong, Ruizhi Zhang, Chenbo Zou, Shenghui Shang, Chi Zhang, Xiangchuang Fan, Lijuan Zhu, Huiquan Duan, Chuan Zhang, Shiqing Feng, Hengxing Zhou

**Affiliations:** ^1^ Department of Orthopaedics, Qilu Hospital of Shandong University, Shandong University Centre for Orthopaedics, Advanced Medical Research Institute, Cheeloo College of Medicine Shandong University Jinan Shandong P. R. China; ^2^ Department of Orthopaedics, The Second Qilu Hospital of Shandong University, Cheeloo College of Medicine Shandong University Jinan Shandong P. R. China; ^3^ School of Chemistry and Chemical Engineering, Frontiers Science Center for Transformative Molecules, Shanghai Key Laboratory for Molecular Engineering of Chiral Drugs Shanghai Jiao Tong University 800 Dongchuan Road Shanghai P. R. China; ^4^ Department of Orthopaedics, Tianjin Medical University General Hospital, Tianjin Medical University International Science and Technology Cooperation Base of Spinal Cord Injury Tianjin Key Laboratory of Spine and Spinal Cord Tianjin P. R. China; ^5^ Department of Ophthalmology, Eye & ENT Hospital, State Key Laboratory of Brain Function and Disorders Fudan University Shanghai P. R. China; ^6^ Institute of Molecular Medicine, Shanghai Jiao Tong University Affiliated Renji Hospital Shanghai Jiao Tong University School of Medicine Shanghai P. R. China; ^7^ Department of Liver Surgery and Transplantation and Key Laboratory of Carcinogenesis and Cancer Invasion, Ministry of Education, Liver Cancer Institute Zhongshan Hospital Fudan University Shanghai P. R. China; ^8^ Department of Geriatric Medicine Qilu Hospital of Shandong University Wenhuaxi Road 107 Jinan Shandong P. R. China

**Keywords:** circuit reconstruction, neuronal reactivation, nucleic acid hydrogel, serotonergic system, spinal cord injury

## Abstract

Despite the persistence of spared spinal circuits capable of relaying commands after spinal cord injury (SCI), their contribution to recovery remains constrained by functional dormancy of spared neurons and impaired reconnection across the lesion. Serotonergic neuromodulation is pivotal for reactivating dormant neurons, however, achieving precise targeting and modulation of the serotonergic system poses translational challenges. Here, a DNA/RNA heteroduplex hydrogel is reported that integrates 5‐hydroxytryptamine (5‐HT)‐mediated neuronal excitability restoration with phosphatase and tensin homolog (PTEN)‐targeted spinal circuit reconstruction for SCI therapy. The 5‐hydroxytryptophan (5‐HTP)‐derived motif, serving both as a targeting ligand and as a neuromodulator, is site‐specifically grafted onto three phosphorothioate‐bearing single‐stranded DNA (ssDNA) strands, which self‐assemble into Y‐shaped motifs and are subsequently crosslinked by sticky‐ended PTEN small interfering RNA (siRNA) to form the hydrogel network. After lesion‐site administration, the hydrogel undergoes DNase‐mediated network disassembly into nanogels that exert two complementary therapeutic actions by targeting serotonergic system: restoring excitability to reactivate dormant interneurons and reconstructing descending connectivity to reintegrate spared circuits with the host spinal cord, thereby restoring sensory and locomotor functions in paralyzed mice. This strategy coordinately reinstates functional excitability and structural rebuilding by engaging multiple interlocking mechanisms, advancing a versatile paradigm for integrative therapy of central nervous system (CNS) disorders.

## Graphical abstract

The study reports a DNA/RNA heteroduplex hydrogel (SeroPTEN‐CG) that undergoes DNase‐mediated hydrogel‐to‐nanogel transition for spinal cord injury therapy by targeting the serotonergic system, combining 5‐hydroxytryptamine (5‐HT)‐mediated excitability restoration to reactivate dormant interneurons with phosphatase and tensin homolog (PTEN)‐targeted spared circuit reconstruction, while shifting inflammatory microglia toward a reparative M2 phenotype to create a neuroimmune microenvironment favorable for functional recovery.

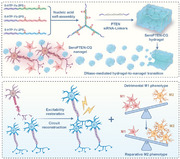

## 1 Introduction

Spinal cord injury (SCI) is a critical central nervous system (CNS) trauma that may result in permanent paralysis, sensory impairments, and autonomic dysfunction [1, 2]. The majority of individuals with SCI sustain anatomically incomplete injuries, leaving spared spinal circuits, primarily composed of local interneurons, capable of serving as relay networks for transmitting commands across the lesion [3, 4, 5]. Nevertheless, functional reintegration of spared circuits is largely constrained by two concurrent mechanisms: substantial disruptions in neuronal excitability that render spared circuits functionally dormant [6, 7, 8], and limited axonal regrowth with defective synaptic reorganization, which together prevent reconnection with the host spinal cord [4, 9, 10]. Although coordinating structural and functional targets may offer a more effective outcome in SCI therapy [2, 11, 12], no single pharmacologic strategy is concurrently capable of reactivating dormant neurons and reconnecting spinal circuits.

Descending commands activate spinal central pattern generators (CPGs) to drive locomotion, while the locomotor parameters are largely governed by monoaminergic neuromodulation, especially serotonin (5‐hydroxytryptamine, 5‐HT) [1, 5, 13]. SCI disrupts serotonergic projections, sharply attenuating 5‐HT concentrations and downstream signaling distal to the lesion [14, 15, 16, 17]. Restoring local 5‐HT amplifies persistent inward currents (PICs) in spinal motoneurons, thereby reactivating dormant spared neurons and facilitating intersegmental synaptic transmission [18, 19, 20]. Despite the above therapeutic promise, current serotonergic intervention strategies remain insufficient to achieve satisfactory efficacy [21]. The widespread distribution of 5‐HT receptors (5‐HTRs) makes systemic adverse effects difficult to avoid [22], and a narrow therapeutic window renders clinical benefit highly dose dependent, thereby constraining the translational portability of clinically established serotonergic pharmacotherapies, such as 5‐HTRs agonists and selective serotonin reuptake inhibitors (SSRIs), in the context of SCI therapy [17, 23, 24, 25]. In response, approaches such as serotonergic progenitor transplantation and electrical stimulation have been developed, however, while transplantation partially restores serotonergic innervation, it carries unresolved issues of donor scarcity and immune rejection [18, 26, 27], whereas electrical or optogenetic stimulation typically requires implanted electrodes or optical hardware, raising safety concerns and restricting patient mobility [28, 29]. Accordingly, a salient challenge is to implement targeted and quantitatively controlled modulation of the serotonergic system at the lesion, which represents a critical step toward deploying serotonergic mechanisms to reactivate dormant spared neurons for SCI therapy.

On the basis of excitability rescue, durable functional recovery requires spinal circuit reconstruction to maintain and relay the restored commands [30, 31, 32], which necessitates not only axonal elongation and synaptic reorganization but also a permissive neuroimmune microenvironment [33, 34]. Within this framework, phosphatase and tensin homolog (PTEN) emerges as a single actionable lever for coordinating neuron‐intrinsic growth programs with neuron‐extrinsic neuroimmune optimization by promoting circuit reconstruction and microglial polarization toward a reparative M2 phenotype [6, 35, 36, 37, 38, 39]. However, PTEN remains an “undruggable” target for conventional protein‐directed inhibitors due to its structurally conserved and electrostatically constrained active site [40, 41, 42], thereby sharpening our attention to sequence‐specific oligonucleotide therapeutics that inaugurate a gene‐targeted era in CNS disorders [43, 44, 45, 46].

To endow oligonucleotide drugs with targeting specificity, researchers have pursued multiple ligand‐conjugation strategies, including peptides, antibodies, and aptamers [47, 48, 49]. Nevertheless, protein ligands increase molecular weight and impede tissue penetration [50, 51], and may elicit anti‐drug antibody responses and complement activation [52, 53], while aptamers still struggle with nuclease stability and binding reliability challenges [54]. These constraints are expanding the field toward ligand classes that harness endogenous trafficking machinery, notably neurotransmitter‐derived small molecules that target transporters or receptors. In practice, such ligand design strategies can convert generic carriers into brain‐permissive vehicles for macromolecules, including oligonucleotide drugs, as shown by neurotransmitter‐derived lipidoids that enable blood‐brain barrier transit and neuronal uptake [55]. Extending this rationale, a representative example incorporates γ‐aminobutyric acid (GABA)‐mimetic ligands with a K^+^‐Cl^−^ co‐transporter (KCC2) activator to target inhibitory interneurons and to normalize intracellular chloride for rebalancing neuronal excitability [8]. Meanwhile, a glutamate‐functionalized nanoconstruct has been developed to target the postsynaptic neurons of glutamatergic synapses for transducing near‐infrared light into localized stimulation [28]. Collectively, these examples motivate us to advance a dual‐function ligand concept in which a single neurotransmitter or precursor‐derived motif serves both as a targeting ligand and as a bioactive neuromodulator, thereby uniting circuit‐specific targeting with excitability modulation in a single molecular design.

**SCHEME 1:**
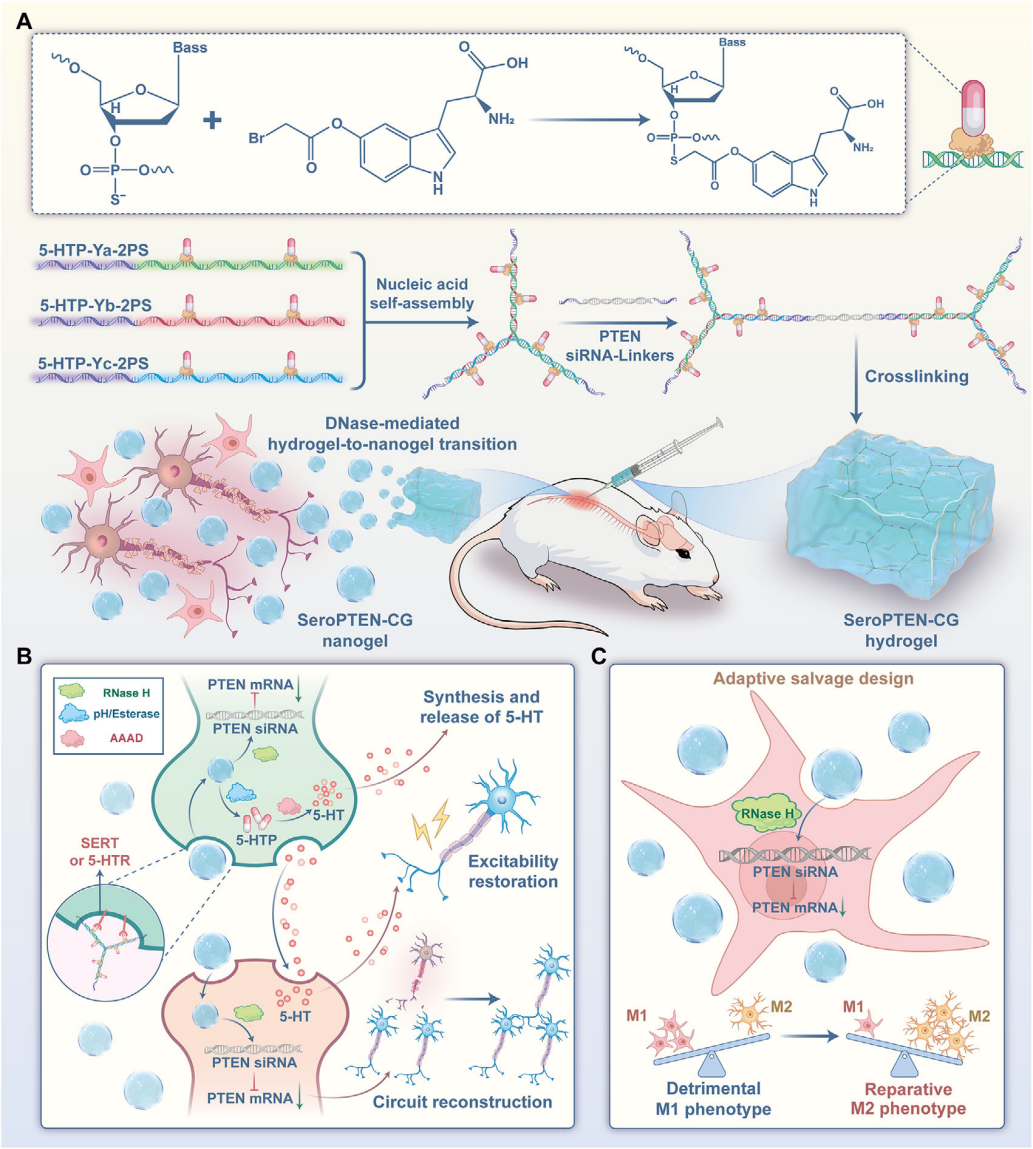
Schematic overview of the self‐assembly and synergistic mechanisms of SeroPTEN‐CG for SCI therapy. (A). Site‐specific conjugation and nucleic acid self‐assembly. Carbonyl bromide‐modified   5‐HTP (5‐HTP‐Br) is covalently grafted onto phosphorothioate (PS) sites on three mutually complementary ssDNA strands to obtain 5‐HTP‐DNA conjugates, which self‐assemble through molecular recognition and base pairing into 5‐HTP‐Y‐motifs. PTEN siRNA‐linkers with complementary sticky ends crosslink the 5‐HTP‐Y‐motifs to generate SeroPTEN‐CG hydrogels with defined 5‐HTP:siRNA payload stoichiometry, which undergo endogenous DNase‐mediated in situ hydrogel‐to‐nanogel transition, generating SeroPTEN‐CG nanogels that penetrate the ECM. (B). Excitability restoration and circuit reconstruction. 5‐HTP‐Br moieties function as serotonergic targeting ligands, directing SeroPTEN‐CG nanogels to target the serotonergic system within spared circuits. Intracellular RNase H cleaves DNA/RNA heteroduplexes, releasing intact PTEN siRNA duplexes that silence *Pten* and activate neuron‐intrinsic growth programs, in parallel, pH/esterase‐mediated hydrolysis of ester linkages liberates 5‐HTP, which is converted to 5‐HT by aromatic L‐amino acid decarboxylase (AADC), reactivating dormant spared neurons. (C). Adaptive salvage design. At the inflammatory lesion site, unavoidable microglial phagocytosis of nanogels is strategically leveraged to drive polarization from a detrimental M1 phenotype toward a reparative M2 phenotype, thereby establishing a neuroimmune microenvironment conducive to SCI therapy. Abbreviations: 5‐HTP, 5‐hydroxytryptophan; 5‐HT, 5‐hydroxytryptamine; PS, phosphorothioate; ssDNA, single‐stranded DNA; siRNA, small interfering RNA; ECM, extracellular matrix; AADC, aromatic L‐amino acid decarboxylase.

To underscore the therapeutic benefit of combining excitability rescue with circuit rebuilding for SCI therapy, we herein propose an integrated pharmacologic strategy that couples reactivation of dormant spared neurons with reconstruction of spinal circuits. As a proof‐of‐concept, we constructed serotonergic system‐targeted DNA/RNA heteroduplex hydrogels, termed SeroPTEN‐Chemogene (SeroPTEN‐CG), achieving stoichiometrically controlled integration of PTEN‐targeted neuron‐intrinsic growth programs with 5‐HT‐mediated excitability restoration (Scheme 1). After lesion‐site administration, the hydrogels undergo DNase‐mediated network disassembly into nanogels that, guided by the 5‐hydroxytryptophan (5‐HTP)‐derived ligand, subsequently target serotonin transporter (SERT)‐expressing serotonergic neurons and 5‐HT receptors (5‐HTRs)‐expressing interneurons within spared circuits [56, 57], where RNase H disassembles the heteroduplex network to release small interfering RNA (siRNA) targeting PTEN for circuit reconstruction [58], while pH/esterase‐mediated cleavage of ester linkages liberates 5‐HTP, the precursor for endogenous 5‐HT synthesis, to reactivate dormant spared neurons [59, 60]. Given the inevitable microglial phagocytosis within inflammatory lesions, an “adaptive salvage design” is further incorporated to repurpose this process for neuroimmune modulation by inducing polarization toward a reparative M2 phenotype, thereby establishing a neuroimmune microenvironment favorable for SCI therapy [61, 62].

Collectively, our oligonucleotide therapeutics strategy integrates 5‐HT‐mediated functional reactivation with PTEN‐targeted structural reconstruction in a single lesion‐site dosing platform, advancing a synergistic paradigm for SCI therapy.

## 2 Results and Discussion

### 2.1 Synthesis and Characterizations of SeroPTEN‐CG

We evaluated multiple indole‐based candidates for constructing a targeted nucleic acid Chemogene platform, and derivatives synthesized from 5‐HTP were ultimately selected due to their dual advantage of mimicking tryptamine architecture to facilitate receptor targeting while serving as biosynthetic precursors for 5‐HT synthesis through AADC‐mediated conversion (Figure S1).

**FIGURE 1:**
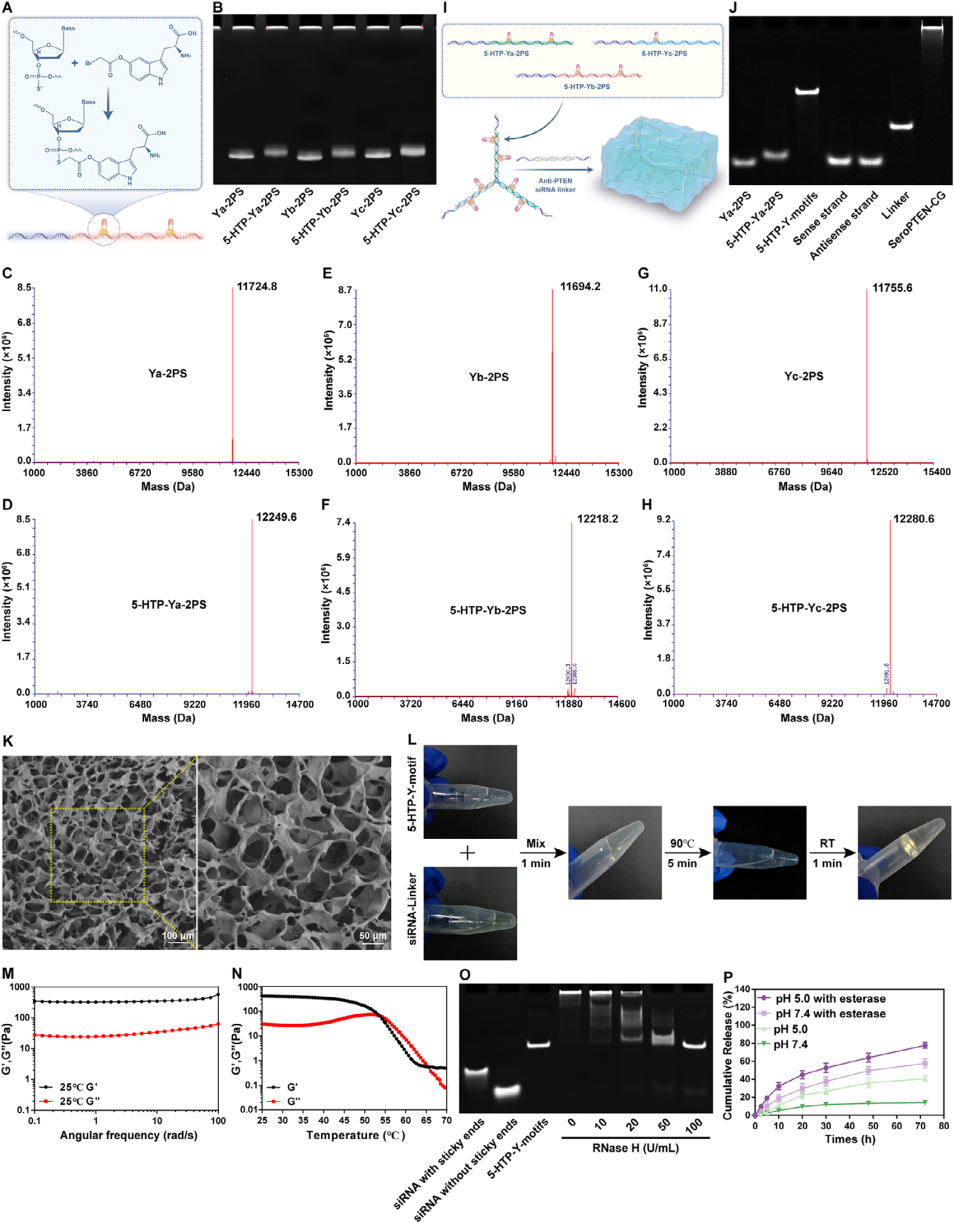
Chemoselective conjugation, self‐assembly, and physicochemical characterization of the SeroPTEN‐CG hydrogels. (A) Site‐selective grafting of 5‐HTP onto two PS modification sites of ssDNA to obtain 5‐HTP‐DNA conjugates. (B) Denaturing PAGE of unmodified ssDNA (Ya‐2PS, Yb‐2PS, Yc‐2PS) and their 5‐HTP conjugates (5‐HTP‐Ya‐2PS, 5‐HTP‐Yb‐2PS, 5‐HTP‐Yc‐2PS). (C–H) Mass shifts consistent with stoichiometric and site‐specific grafting, indicating full occupancy of two PS modification sites. (I) Self‐assembly scheme: 5‐HTP‐Y‐motifs crosslinked by PTEN siRNA‐linkers to form SeroPTEN‐CG hydrogels. (J) 10% native PAGE verifying self‐assembly of 5‐HTP‐Y‐motifs and PTEN siRNA‐linkers, and the SeroPTEN‐CG hydrogels barely migrated into the gel matrix. (K) SEM of the freeze‐dried SeroPTEN‐CG hydrogels showing an interconnected porous network. (L) Photographs illustrating rapid and reversible thermo‐responsive behavior: gelation upon mixing at room temperature, liquefaction at 90°C (5 min), and re‐gelation after cooling to room temperature (1 min). (M) Frequency sweeps showing storage modulus (G′) consistently exceeding loss modulus (G″), characteristic of stable hydrogels. (N) Temperature sweep showing progressive decrease of G′ with increasing temperature and a G′/G″ crossover at approximately 55°C. (O) 10% native PAGE demonstrating RNase H‐mediated release of PTEN siRNA from SeroPTEN‐CG hydrogels, and band intensity increases with RNase H (0‐100 U/mL). (P) Cumulative 5‐HTP release under indicated conditions: minimal release at pH 7.4 (<20% within 72 h), enhanced release at pH 5.0 (∼40% within 72 h), and synergistically accelerated at pH 5.0 with esterase (17 U/mL; >70% within 72 h). Scale bar, as indicated. Abbreviations: 5‐HTP, 5‐hydroxytryptophan; PS, phosphorothioate; ssDNA, single‐stranded DNA; PAGE, polyacrylamide gel electrophoresis; SEM, scanning electron microscopy; G′, storage modulus; G″, loss modulus.

For covalent conjugation of 5‐HTP to ssDNA, 5‐HTP was functionalized to its carbonyl bromide‐modified derivative (5‐HTP‐Br, compound 3) through a three‐step synthetic procedure (Figure S2), providing a reactive handle for oligonucleotide conjugation while retaining the indoleamine pharmacophore and permitting AADC‐mediated conversion. The chemical structures of all intermediates and the final compound were confirmed by proton nuclear magnetic resonance (^1^H NMR) and carbon nuclear magnetic resonance (^13^C NMR), with spectral peaks consistent with the predicted structures (Figure S3–S8). Mass spectrometry (MS) provided complementary confirmation, with measured m/z matching theoretical calculations, further verifying the successful synthesis of 5‐HTP‐Br (Figure S9). For conjugation to oligonucleotides, as a proof‐of‐concept, 5‐HTP‐Br was coupled to two PS modification sites dispersedly designed on each of three mutually complementary ssDNA (Ya‐2PS, Yb‐2PS, Yc‐2PS; Table S1, Supporting Information) to obtain 5‐HTP‐DNA conjugates (5‐HTP‐Ya‐2PS, 5‐HTP‐Yb‐2PS, 5‐HTP‐Yc‐2PS) (Figure 1A). These conjugates were confirmed by 15% denaturing polyacrylamide gel electrophoresis (PAGE), which revealed sharp, monodisperse bands migrating more slowly than unmodified ssDNA (Figure 1B). Consistently, the masses of unconjugated Ya‐2PS, Yb‐2PS, and Yc‐2PS were 11724.8, 11694.2, and 11755.6 Da, which increased to 12249.6, 12218.2, and 12280.6 Da after 5‐HTP conjugation, respectively, confirming the successful synthesis of 5‐HTP‐DNA conjugates (Figure 1C–H). The successful self‐assembly of 5‐HTP‐Y‐motifs and PTEN siRNA‐linkers into SeroPTEN‐CG hydrogels was confirmed by 10% native PAGE, evidenced by sharp and monodisperse bands migrating more slowly than their component strands, whereas the assembled SeroPTEN‐CG hydrogels barely migrated into the gel matrix (Figure 1I,J). Scanning electron microscopy (SEM) further revealed a characteristic porous microstructure, providing evidence of effective crosslinking between 5‐HTP‐Y‐motifs and PTEN siRNA‐linkers at the microscale (Figure 1K).

The SeroPTEN‐CG hydrogels exhibited reversible thermo‐responsive gel‐to‐sol transitions, liquefying on heating to 90°C for 5 minutes (min) and spontaneously reverting to original gel state within 1 min at room temperature (Figure 1L), consistent with disruption and reformation of Watson‐Crick base pairing mediated crosslinks between nucleic acid building blocks, thereby providing a convenient working window for intraoperative handling, including loading, injection, and in situ positioning within the lesion cavity. The properties of the hydrogels were further characterized using a rotational rheometer, and frequency‐dependent rheology indicated that the storage modulus (G′) consistently surpassed the loss modulus (G″) across the entire frequency range, confirming typical hydrogel behavior and providing a mechanical baseline for subsequent thermal evaluation (Figure 1M). Temperature‐dependent rheology revealed a gradual decrease in G′ from 25 to 70°C, with G′ intersecting G″ at approximately 55°C (Figure 1N), suggesting that the hydrogel integrity is dictated by molecular recognition and complementary base pairing, as thermal disruption of hybridization‐mediated crosslinks triggers disassembly of the network structure.

The SeroPTEN‐CG hydrogels are assembled through crosslinking between 5‐HTP‐Y‐motifs and PTEN siRNA‐linkers, forming DNA/RNA heteroduplexes whose RNA strands can be selectively cleaved by RNase H to release functional PTEN siRNA. To verify this RNase H‐mediated release mechanism, SeroPTEN‐CG hydrogels were incubated with increasing RNase H concentrations before being analyzed by 10% native PAGE, which revealed a distinct band corresponding to the PTEN siRNA, with intensity increasing proportionally, confirming RNase H‐dependent release of PTEN siRNA (Figure 1O). In parallel, 5‐HTP is conjugated to PS sites of ssDNA through ester bonds, whose hydrolysis is susceptible to pH and esterase. The release kinetics of 5‐HTP were measured under different pH conditions (neutral pH 7.4 or acidic pH 5.0), either with or without esterase (17 U/mL), followed by quantitative analysis using high‐performance liquid chromatography (HPLC). Under physiological conditions (pH 7.4), the SeroPTEN‐CG exhibited limited 5‐HTP release (<20%) within 72 hours (h), however, lowering the pH to acidic conditions increased the release to approximately 40%, and the simultaneous exposure to enzymatic conditions produced a synergistic effect, resulting in >70% cumulative release within the same period (Figure 1P), confirming extracellular stability as a targeting ligand while enabling rapid intracellular release as a neurotransmitter precursor.

The site‐specific conjugation of 5‐HTP‐Br to PS sites, together with assembly mediated by complementary base pairing, establishes defined payload stoichiometries with quantitative 5‐HTP:siRNA ratios. Additionally, two independent triggers, RNase H‐mediated release of siRNA and pH/esterase‐mediated release of 5‐HTP, produce decoupled release kinetics that diminish mutual interference and permit stoichiometric titration for synergistic therapy, supporting the designed combination of excitability rescue and circuit rebuilding for SCI therapy.

### 2.2 Serotonergic System Targeting and Lesion‐site Localization

**FIGURE 2:**
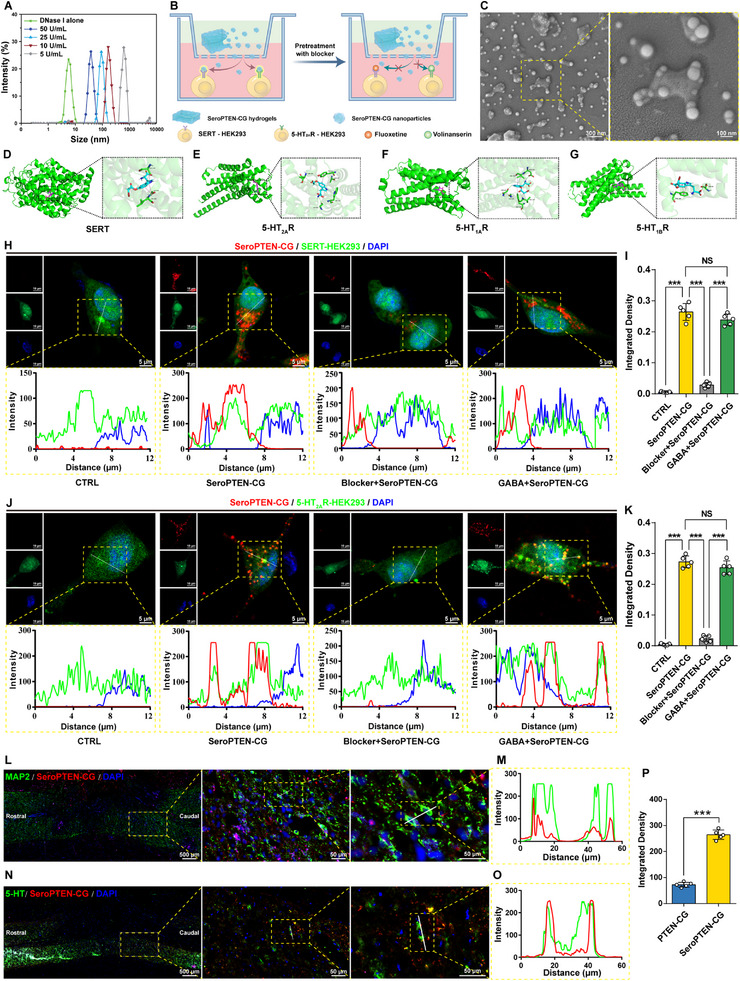
DNase‐mediated hydrogel‐to‐nanogel transition and receptor‐directed targeting of SeroPTEN‐CG. (A). DLS size distributions of nanogels released from SeroPTEN‐CG hydrogels after incubation with DNase I (5–50 U/mL) show progressive size reduction with increasing enzyme. (B). Transwell assay schematic shows SeroPTEN‐CG hydrogels in the upper chamber and SERT‐HEK293 or 5‐HT_2A_R‐HEK293 cells in the lower chamber, with pretreatment using Fluoxetine (SERT blocker) or Volinanserin (5‐HT_2A_R blocker). (C). SEM images of nanogels in the supernatant show uniformly spherical morphology with favorable dispersibility (right: higher magnification). (D‐G). Molecular docking of 5‐HTP‐Br shows that the lowest‐energy poses are localized in extracellularly accessible pockets of SERT (D), 5‐HT_2A_R (E), 5‐HT_1A_R (F), and 5‐HT_1B_R (G), with representative noncovalent interactions. (H). Confocal images show colocalization between SeroPTEN‐CG (red) and SERT‐HEK293 cells (green), with representative line‐scan profiles displayed (bottom). (I). Quantification of integrated density on SERT‐HEK293 cells (n  = 5 per group). (J, K). The same analysis is shown for 5‐HT_2A_R‐HEK293 cells, with Volinanserin blocking receptor‐dependent colocalization (n  = 5 per group). (L, M). In vivo confocal images in mouse spinal cord hemisection models show colocalization of Cy5.5‐SeroPTEN‐CG (red) with MAP2⁺ neurons (green) at lesion site, with representative line‐scan profiles displayed (right). (N, O) In vivo confocal images and representative line‐scan profiles show colocalization of Cy5.5‐SeroPTEN‐CG (red) with 5‐HT⁺ neurons (green). (P). Quantification of Cy5.5 signal within lesions indicates higher accumulation for SeroPTEN‐CG than PTEN‐CG (n  = 5 per group). Scale bar, as indicated. Quantitative data in (I, K, P) are presented as mean ± standard error of the mean (SEM). Statistical significance is assessed using one‐way ANOVA with Tukey's post hoc test for multiple comparisons. ^***^ *p* < 0.001; NS, not significant. Abbreviations: DLS, dynamic light scattering; SERT, serotonin transporter; MAP2, microtubule‐associated protein 2; 5‐HT, 5‐hydroxytryptamine; DAPI, 4′,6‐diamidino‐2‐phenylindole.

We hypothesized that the SeroPTEN‐CG hydrogels would undergo enzymatic cleavage to form nanogels after administration to the lesion site. To validate this, hydrogels were incubated with increasing concentrations of DNase I at 37°C for 12 h, after which the supernatants were collected, and the particle size distributions were analyzed by dynamic light scattering (DLS). As shown in Figure 2A, the resulting nanogel size progressively decreased with increasing DNase I concentration, indicating a DNase‐mediated disassembly profile of SeroPTEN‐CG hydrogels that supports lesion‐site nanogel formation.

To evaluate the targeting capability of SeroPTEN‐CG nanogels under simulated physiological conditions, a Transwell co‐culture system was established: SeroPTEN‐CG hydrogels were loaded into the upper chamber, whereas SERT‑HEK293 cells (HEK293 cells engineered to express SERT) and 5‐HT_2A_R‑HEK293 cells (HEK293 cells engineered to express 5‐HT_2A_R) were cultured in the lower chamber (Figure 2B). After incubation at 37°C for 12 h to allow nanogels to diffuse across the porous membrane and interact with the underlying target cells, the supernatants were collected for SEM analysis, which revealed that nanogels exhibited a uniformly spherical morphology with favorable dispersibility, suggesting the potential for efficient diffusion and cell targeting (Figure 2C).

Given that 5‐HTP‐Br is designed to serve as a targeting ligand, we next investigated the molecular basis of receptor‐specific interactions. To capture the major presynaptic and postsynaptic serotonergic modulatory sites within spinal locomotor circuits, molecular docking was performed against SERT, 5‐HT_2A_R, 5‐HT_1A_R, and 5‐HT_1B_R, which collectively represent targets within CPGs and propriospinal interneuron networks implicated in locomotor recovery after SCI [18, 20, 63, 64]. Molecular docking simulations indicated the binding free energies were −7.066 kcal/mol for SERT and −7.252 kcal/mol for 5‐HT_2A_R, reflecting spontaneous and energetically favorable interactions. The docking conformations of 5‐HTP‐Br with each receptor in the lowest‐energy state were extracted for structural analysis (Figure 2D,E). On SERT, 5‐HTP‐Br occupied an extracellularly accessible binding pocket and formed multiple noncovalent contacts with amino acid residues including ALA‐169, GLY‐442, and LEU‐443 in the vicinity of the binding site (Figure S10). In the case of 5‐HT_2A_R, 5‐HTP‐Br similarly occupied an extracellularly accessible binding pocket, forming multiple noncovalent interactions with amino acid residues (such as ASP‐155, VAL‐156, and PHE‐340) (Figure S11). Extending this analysis to 5‐HT_1A_R and 5‐HT_1B_R revealed analogous extracellular surface‐accessible pockets and binding free energies comparable to those observed in SERT and 5‐HT_2A_R (Figure 2F,G), and the predicted conformations were stabilized by analogous noncovalent interactions (Figures S12 and S13), indicating that 5‐HTP‐Br can interact with SERT and multiple 5‐HTRs subtypes, consistent with broad‐spectrum serotonergic affinity and validating its design rationale as the targeting moiety.

Consistent with molecular docking simulations, confocal imaging demonstrated significant fluorescence colocalization between Cy5.5‐labeled SeroPTEN‐CG nanogels and both SERT‑HEK293 and 5‑HT_2A_R‑HEK293 cells, with line‐scan profiles revealing overlapping signals within the cytoplasm, suggesting efficient ligand‐receptor recognition and targeting specificity conferred by 5‐HTP‐Br modification. Moreover, pretreatment with Fluoxetine (a SERT blocker) or Volinanserin (a 5‐HT_2A_R blocker) significantly diminished colocalization, while pretreatment with GABA, employed as a negative control, exhibited no significant impact (Figure 2H,J). Quantitative fluorescence intensity further revealed that the SeroPTEN‐CG group exhibited the highest integrated fluorescence among tested conditions, consistent with the greatest nanogels colocalization with target cells (Figure 2I,K). Together, these results demonstrate that 5‐HTP‐Br modification endows SeroPTEN‐CG nanogels with high‐affinity and receptor‐dependent targeting toward both SERT and 5‐HTRs in vitro.

We next asked whether this targeting capability persists in vivo within the complex microenvironment of the lesion site. Following local administration of SeroPTEN‐CG hydrogels and subsequent disassembly for 24 h, spinal cord tissues were collected for fluorescence colocalization analysis. The red fluorescence of SeroPTEN‐CG nanogels accumulated selectively within the injured region and showed substantial colocalization with microtubule‐associated protein 2 (MAP2)‐positive (MAP2^+^) mature neurons (Figure 2L,M), which represent neuronal populations spared from the primary injury and contribute to reestablishing functional signal relay. Concurrent colocalization with 5‐HT‐positive (5‐HT^+^) neurons confirmed efficient targeting of serotonergic populations that modulate locomotor network reactivation (Figure 2N,O). In contrast, negligible accumulation and colocalization were detected in PTEN‐CG (PTEN siRNA Chemogene, without 5‐HTP‐Br modification) group (Figure S14), and quantitative analysis showed significantly higher fluorescence intensity for SeroPTEN‐CG than PTEN‐CG within lesions (Figure 2P), highlighting the necessity of 5‐HTP‐Br for mediating receptor‐specific targeting and retention at the lesion site, thereby reducing clearance and prolonging local persistence.

Taken together, these results demonstrate that SeroPTEN‐CG hydrogels undergo DNase‐mediated disassembly into nanogels that subsequently penetrate the extracellular matrix (ECM) and target the serotonergic system through receptor‐dependent recognition. This two‐phase sequence establishes a therapeutic paradigm that enables enriched delivery to both the excitability module and the neuron‐intrinsic growth programs while limiting systemic adverse effects.

### 2.3 Neuronal PTEN Silencing and Axonal Elongation

**FIGURE 3:**
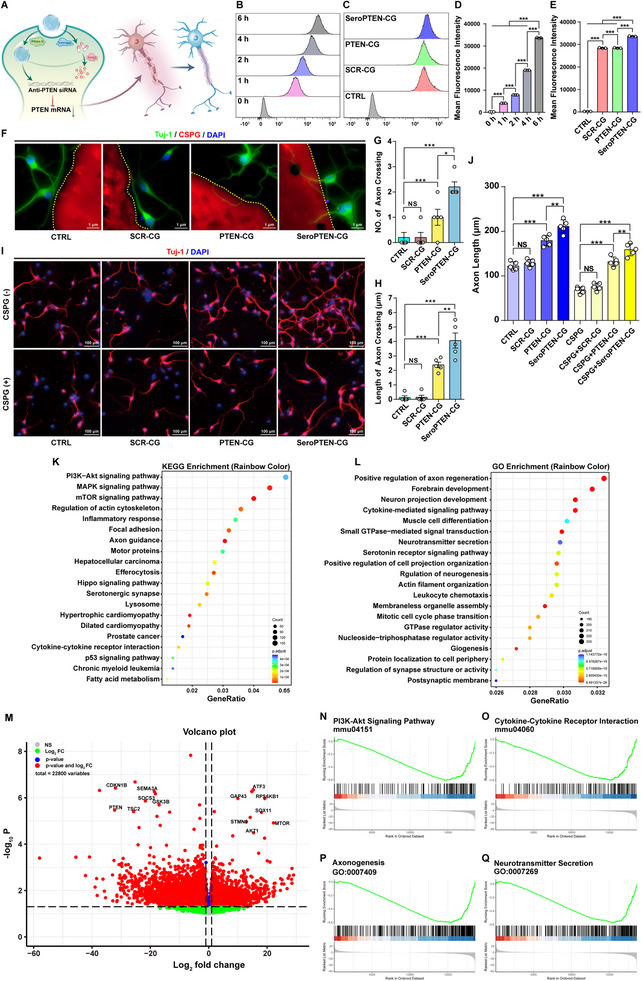
Neuronal uptake, PTEN silencing, axonal elongation, and bioinformatics analysis after SeroPTEN‐CG treatment. (A) The schematic illustrates neuronal internalization of SeroPTEN‐CG, RNase H‐mediated release of PTEN siRNA, and subsequent axonal elongation. (B) Time‐course FCM analysis of neurons incubated with FAM‐labeled SeroPTEN‐CG shows progressive rightward shifts. (C) FCM analysis of neurons incubated with FAM‐labeled formulations (SCR‐CG, PTEN‐CG, SeroPTEN‐CG) at 6 h. (D) Quantification of MFI over time demonstrates time‐dependent internalization (n  = 3 per group). (E) Quantification of MFI at 6 h across groups shows that SeroPTEN‐CG group exhibits the highest intracellular signal (n  = 3 per group). (F) The axonal guidance spot assay across CSPG barriers (CSPG, red; Tuj1⁺ axons, green) shows increased crossings with SeroPTEN‐CG treatment. Dashed lines mark CSPG interfaces. (G, H) Quantification of axonal crossings per neuron (G) and mean crossing length (H); SeroPTEN‐CG significantly increases both metrics relative to other groups (n  = 5 per group). (I) Axonal elongation under diffuse CSPG⁺ or CSPG^−^ conditions with the formulations (SCR‐CG, PTEN‐CG, SeroPTEN‐CG). (J) Axon‐length quantification under CSPG^−^ or CSPG⁺ conditions shows that CSPG reduces the length of axons, whereas SeroPTEN‐CG restores elongation (n  = 5 per group). (K, L) KEGG (K) and GO (L) enrichment for DEGs after treatment highlight the pathways or processes related to PI3K‐Akt/mTOR signaling, cytoskeleton regulation, axon guidance or regeneration, and inflammatory responses. (M) Volcano plot displays transcriptomic changes with selected genes annotated. (N‐Q) GSEA enrichment plots show positive enrichment of PI3K‐Akt signaling pathway (N), cytokine‐cytokine receptor interaction (O), axonogenesis (P), and neurotransmitter secretion (Q) after SeroPTEN‐CG treatment. Scale bar, as indicated. Quantitative data in (D, E, G, H, J) are presented as mean ± SEM. Statistical significance is assessed using one‐way ANOVA with Tukey's post hoc test for multiple comparisons. ^*^ *p* < 0.05, ^**^ *p* < 0.01, ^***^ *p* < 0.001; NS, not significant. Abbreviations: MFI, mean fluorescence intensity; FCM, flow cytometry; CSPG, chondroitin sulfate proteoglycan; DEGs, differentially expressed genes; GO, Gene Ontology; KEGG, Kyoto Encyclopedia of Genes and Genomes; GSEA, Gene Set Enrichment Analysis.

Building on receptor‐directed targeting, we examined whether SeroPTEN‐CG could be efficiently internalized by neurons to promote axonal elongation (Figure 3A). Neurons were incubated with FAM‐labeled SeroPTEN‐CG for 0, 1, 2, 4, or 6 h, and flow cytometry (FCM) analysis demonstrated a time‐dependent increase in intracellular mean fluorescence intensity (MFI) (Figure 3B,D), indicating rapid neuronal uptake and cytoplasmic accumulation of SeroPTEN‐CG nanogels, establishing the basis for intracellular siRNA delivery. To compare neuronal internalization efficiency, intracellular MFI at 6 h was quantified across SCR‐CG (Scrambled siRNA Chemogene, without 5‐HTP‐Br modification), PTEN‐CG, and SeroPTEN‐CG groups. The SeroPTEN‐CG group exhibited the highest MFI compared with SCR‐CG and PTEN‐CG groups (Figure 3C,E), which may be attributed to the ligand‐mediated enhancement of targeting specificity and binding affinity, underscoring the necessity of integrating receptor‐targeting motifs [65, 66].

To determine whether internalization translated into PTEN silencing, neurons were treated with phosphate‐buffered saline (PBS), SCR‐CG, PTEN‐CG, or SeroPTEN‐CG, and subjected to quantitative real‐time PCR (qRT‐PCR) and western blot analysis. Both PTEN‐CG and SeroPTEN‐CG significantly induced downregulation of PTEN messenger RNA (mRNA), remarkably, the SeroPTEN‐CG group exhibited the most pronounced suppression, consistent with its superior neuronal internalization (Figure S15A). Consistently, western blot confirmed the largest decrease in PTEN protein in the SeroPTEN‐CG group (Figure S15B, C), underscoring prominent silencing efficacy at both transcriptional and translational levels. Collectively, these results demonstrate that SeroPTEN‐CG achieves superior PTEN silencing, thereby establishing a mechanistic foundation for the reconstruction of spinal circuits.

To investigate whether SeroPTEN‐CG could activate neuron‐intrinsic programs to overcome the inhibitory barrier imposed by scar components, we evaluated its ability to counteract axonal elongation suppression mediated by chondroitin sulfate proteoglycan (CSPG), the principal inhibitory constituent of post‐injury scar tissue [67]. In the axonal guidance spot assay, SeroPTEN‐CG markedly increased axonal penetration across the CSPG barrier, with mean numbers of crossings exceeding 2 per neuron and mean crossing length surpassing 4 µm, both significantly higher than all other groups (Figure 3F‐H). To validate these results under more diffuse inhibitory conditions, neurons were cultured in CSPG‐containing (CSPG⁺) or CSPG‐free (CSPG^−^) medium supplemented with four formulations (Figure 3I). Consistently, CSPG exposure markedly reduced axonal length relative to CSPG^−^ controls, whereas SeroPTEN‐CG significantly restored axonal elongation under both CSPG⁺ and CSPG^−^ conditions (Figure 3J). Altogether, these results demonstrate that SeroPTEN‐CG effectively attenuates CSPG‐mediated inhibition and promotes axonal elongation, providing a mechanistic basis and translational potential for reestablishing functional neural circuits after SCI [68].

Encouraged by these results, we next compared transcriptome data before and after SeroPTEN‐CG treatment to identify differentially expressed genes (DEGs) and associated signaling pathways underlying neuroregeneration. Kyoto Encyclopedia of Genes and Genomes (KEGG) and Gene Ontology (GO) enrichment analyses were conducted to explore the biological functions and pathways involved. The top 20 KEGG pathways included PI3K‐Akt, MAPK, mTOR signaling pathway, regulation of actin cytoskeleton, and inflammatory response (Figure 3K). GO analysis revealed enrichment in processes such as positive regulation of axonal regeneration, neuron projection development, and cytokine‐mediated signaling (Figure 3L). DEGs were identified between the two groups and visualized in a volcano plot, with representative genes labeled (Figure 3M). Additionally, Gene Set Enrichment Analysis (GSEA) was performed using the hallmark gene set database, further highlighting positive enrichment of PI3K‐Akt signaling pathway (Figure 3N), cytokine‐cytokine receptor interaction (Figure 3O), axonogenesis (Figure 3P), and neurotransmitter secretion (Figure 3Q). These findings indicate that the intervention significantly remodels signaling pathways involved in neuroregeneration, injury repair, and inflammation.

The convergence of neuronal uptake, PTEN silencing, axonal elongation, and enrichment of pro‐regenerative pathways supports a coherent mechanism: receptor‐mediated intracellular delivery of SeroPTEN‐CG activates neuron‐intrinsic PI3K‐Akt/mTOR‐associated growth programs and promotes axonal elongation. Moreover, the transcriptomic enrichment for regulation of the actin cytoskeleton and axonogenesis provides an integrated mechanistic basis for reconstructing descending connectivity.

### 2.4 Neuroimmune Modulation and Neuroprotection

SCI initiates a multifaceted pathological cascade, beginning immediately with primary injury followed by an inflammation‐driven secondary phase [11], which is largely dominated by M1 phenotype microglia whose sustained pro‐inflammatory cytokine release exacerbates neurotoxicity, damages spared circuits, and suppresses regenerative signaling [69]. Reprogramming microglia toward a reparative M2 phenotype has been recognized as an essential strategy to interrupt these cascades and establish a pro‐regenerative neuroimmune microenvironment [61, 70]. Building on this rationale, we advance an “adaptive salvage design”, an underexplored therapeutic strategy that exploits the intrinsic phagocytic activity of M1 microglia to induce their conversion to the reparative M2 phenotype (Figure S16A).

We first examined the silencing efficiency of PTEN expression after nanogel internalization. After 24 h of incubation, western blot analysis revealed significant downregulation of PTEN expression in microglia treated with either SeroPTEN‐CG or PTEN‐CG compared to control (CTRL) and SCR‐CG groups (Figure S16B,C), confirming effective gene silencing mediated by both formulations. Notably, in contrast to differential effects observed in neurons, no significant difference was detected between PTEN‐CG and SeroPTEN‐CG in microglia, a plausible explanation is that the efficient internalization of SeroPTEN‐CG by microglia is dominated by their intrinsic phagocytic capacity. Then, lipopolysaccharide (LPS) primed microglia were incubated with SCR‐CG, PTEN‐CG, or SeroPTEN‐CG for 24 h before immunofluorescence analysis (Figure S16D,E). Compared to the CTRL and SCR‐CG groups, both PTEN‐CG and SeroPTEN‐CG significantly downregulated inducible nitric oxide synthase (iNOS) intensity and the iNOS/ionized calcium binding adapter molecule 1 (Iba1) index while increasing arginase 1 (Arg1) intensity and the Arg1/Iba1 index (Figure S16F–I), indicating a pronounced phenotypic reprogramming from detrimental M1 toward M2 phenotype. This reprogramming was further corroborated by qRT‐PCR, which showed significant iNOS transcript suppression and Arg1 transcript induction in both PTEN‐CG and SeroPTEN‐CG groups compared with CTRL and SCR‐CG groups, with no difference between the two PTEN‐based Chemogene formulations (Figure S16J, K).

**FIGURE 4:**
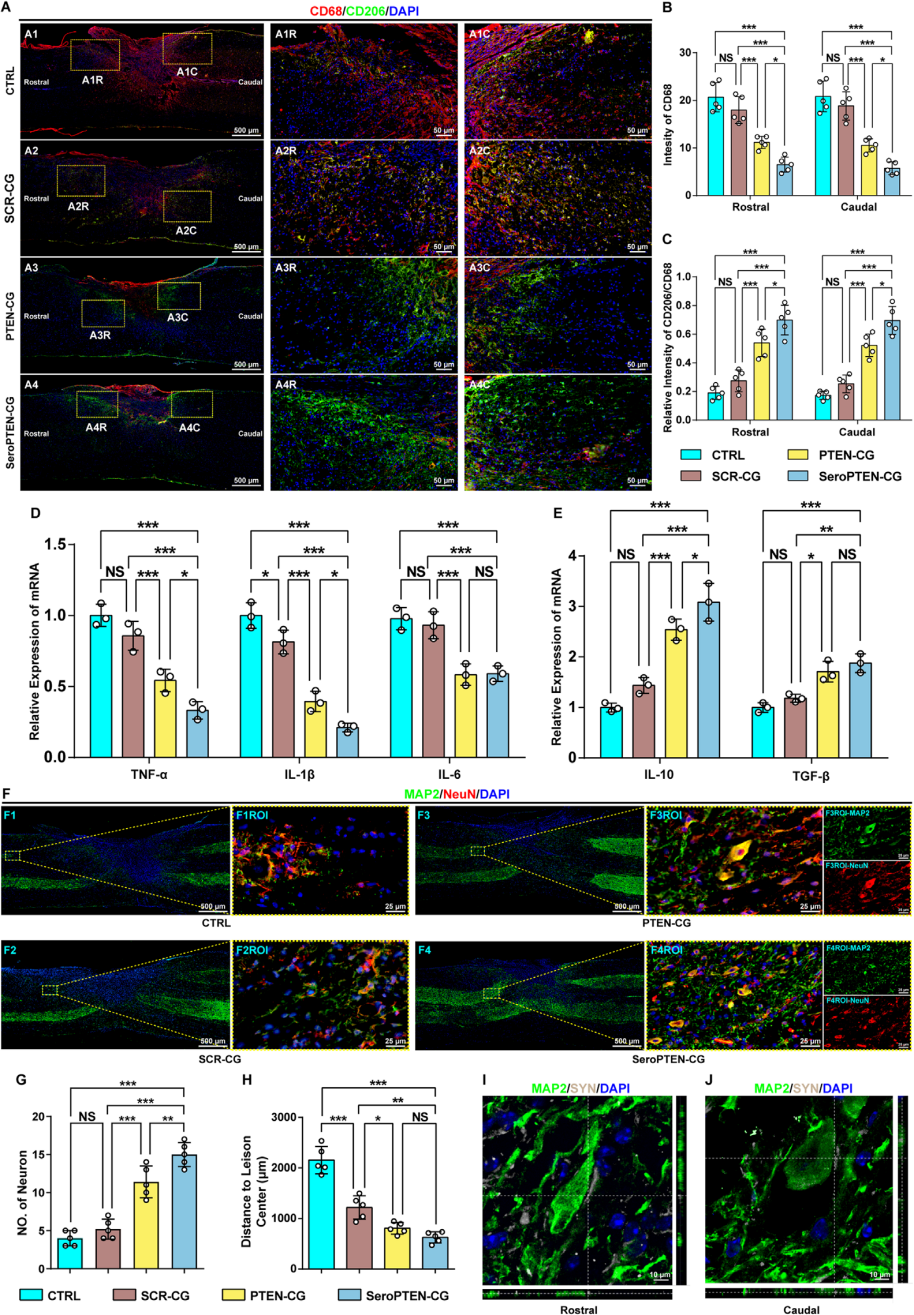
SeroPTEN‐CG reprograms microglia toward a reparative M2 phenotype for neuroimmune modulation and neuroprotection. (A) In vivo immunostaining at 2 weeks post‐SCI shows CD68 (red) and CD206 (green) across different groups (CTRL, SCR‐CG, PTEN‐CG, SeroPTEN‐CG), with boxed rostral (R) and caudal (C) regions displayed at higher magnification. (B, C) Quantification of CD68 intensity (B) and the CD206/CD68 index (C) in rostral and caudal regions (n = 5 per group). (D, E) Cytokine mRNA profiles by qRT‐PCR from injured cords show reduced pro‐inflammatory (TNF‐α, IL‐1β, IL‐6) (D) and increased anti‐inflammatory (IL‐10, TGF‐β) (E) after SeroPTEN‐CG treatment (n = 3 per group). (F) Representative peri‐lesional images from CTRL, SCR‐CG, PTEN‐CG, and SeroPTEN‐CG groups show MAP2 (green) and NeuN (red). Right panels (F1ROI‐F4ROI) are higher‐magnification views of the boxed regions. (G, H) Quantifications show the density of interneurons within the peri‐lesional ROI (G) and the mean distance of spared interneurons from the lesion center (H). Both metrics indicate neuroprotection with PTEN‐CG and a greater effect with SeroPTEN‐CG (n = 5 per group). (I, J) High‐magnification images demonstrate preservation of synaptic architecture: SYN (gray) colocalizes with MAP2⁺ neurons (green) at rostral (I) and caudal (J) lesion sites. Scale bar, as indicated. Quantitative data in (B‐E, G, H) are presented as mean ± SEM. Statistical significance is assessed using one‐way ANOVA with Tukey's post hoc test for multiple comparisons. ^*^ *p* < 0.05, ^**^ *p* < 0.01, ^***^ *p* < 0.001; NS, not significant. Abbreviations: ROI, region of interest; MAP2, microtubule‐associated protein 2; NeuN, neuronal nuclear antigen; SYN, synaptophysin.

Building on the in vitro evidence, we proceeded to investigate microglial activation (CD68 intensity) and polarization (CD206/CD68 index) in a mouse SCI model at 2 weeks post‐injury, a pivotal timepoint characterized by peak microglial activation and infiltration that exacerbate secondary injury (Figure 4A) [36, 71]. Both the CTRL and SCR‐CG groups exhibited elevated CD68 intensity with minimal CD206 co‐expression, indicative of a predominantly pro‐inflammatory M1 phenotype. By contrast, SeroPTEN‐CG substantially reduced CD68 intensity while increasing the proportion of CD68⁺/CD206⁺ double‐positive microglia (Figure 4B,C), indicating pronounced polarization toward the reparative M2 phenotype. Notably, while PTEN‐CG achieved comparable microglial polarization in vitro, SeroPTEN‐CG achieved superior efficacy in vivo (Figure 4B,C), which may be attributed to receptor‐assisted tissue retention and bioavailability of SeroPTEN‐CG at the lesion site. Consistently, the neuroimmune modulation was further substantiated by qRT‐PCR, as the expression levels of classical pro‐inflammatory cytokines [tumor necrosis factor‐α (TNF‐α), interleukin‐1β (IL‐1β), and IL‐6] were significantly downregulated with concomitant upregulation of anti‐inflammatory cytokines [IL‐10 and transforming growth factor‐β (TGF‐β)] in the SeroPTEN‐CG group compared to other groups (Figure 4D, E).

The optimized neuroimmune microenvironment mitigates neurotoxicity and secondary injury, thereby preserving spared neuronal and synaptic integrity, which in turn serves as the structural and functional substrate for subsequent reconstruction of spinal circuits [72, 73]. As shown in Figure 4F, both PTEN‐CG and SeroPTEN‐CG groups conferred significant neuroprotection and improved neuronal survival, as evidenced by increased interneuron density compared to other groups (Figure 4G). In particular, further enhancement with SeroPTEN‐CG treatment presumably resulted from the synergistic benefits of targeting specificity and neuronal PTEN suppression, thereby activating neuron‐intrinsic PI3K‐Akt/mTOR signaling pathway, a mechanism recently recognized as critical for neuroprotection beyond its established roles in axonal regeneration and synaptic remodeling [74]. In parallel with improved neuronal survival, both PTEN‐CG and SeroPTEN‐CG treatments markedly shortened the average distance of spared interneurons from the lesion center (Figure 4H), indicating spatial containment of injury‐induced neuronal loss. Such spatial preservation is particularly important, as neurons closer to the lesion center better support functional integration of reconstituted circuits [75]. Morphological analysis further revealed that protected spared neurons retained mature phenotypic characteristics and colocalized with synaptophysin (SYN), suggesting preservation of functional synaptic architecture and potential signal transduction capability (Figure 4I,J).

Current anti‐inflammatory strategies in SCI therapy are limited by systemic exposure and therapeutic windows [76, 77]. This work exploits the inevitability of microglial phagocytosis as a programmable entry point to deliver PTEN siRNA into microglia, converting off‐target uptake into on‐target immunoreprogramming to promote M2 polarization. Moreover, because this salvage relies on phagocytic uptake rather than receptor targeting, it is intrinsically robust to target heterogeneity in inflamed tissue and conceptually extends beyond SCI to other injury contexts. Collectively, this strategy helps mitigate the persistent problem of payload loss from innate clearance and introduces a rarely explored route for inflammation control, in which phagocytosis is not just an obstacle to be avoided but a design variable to be harnessed for durable neuroimmune modulation.

### 2.5 ECM Remodeling and Specific Spinal Circuits Integration

**FIGURE 5:**
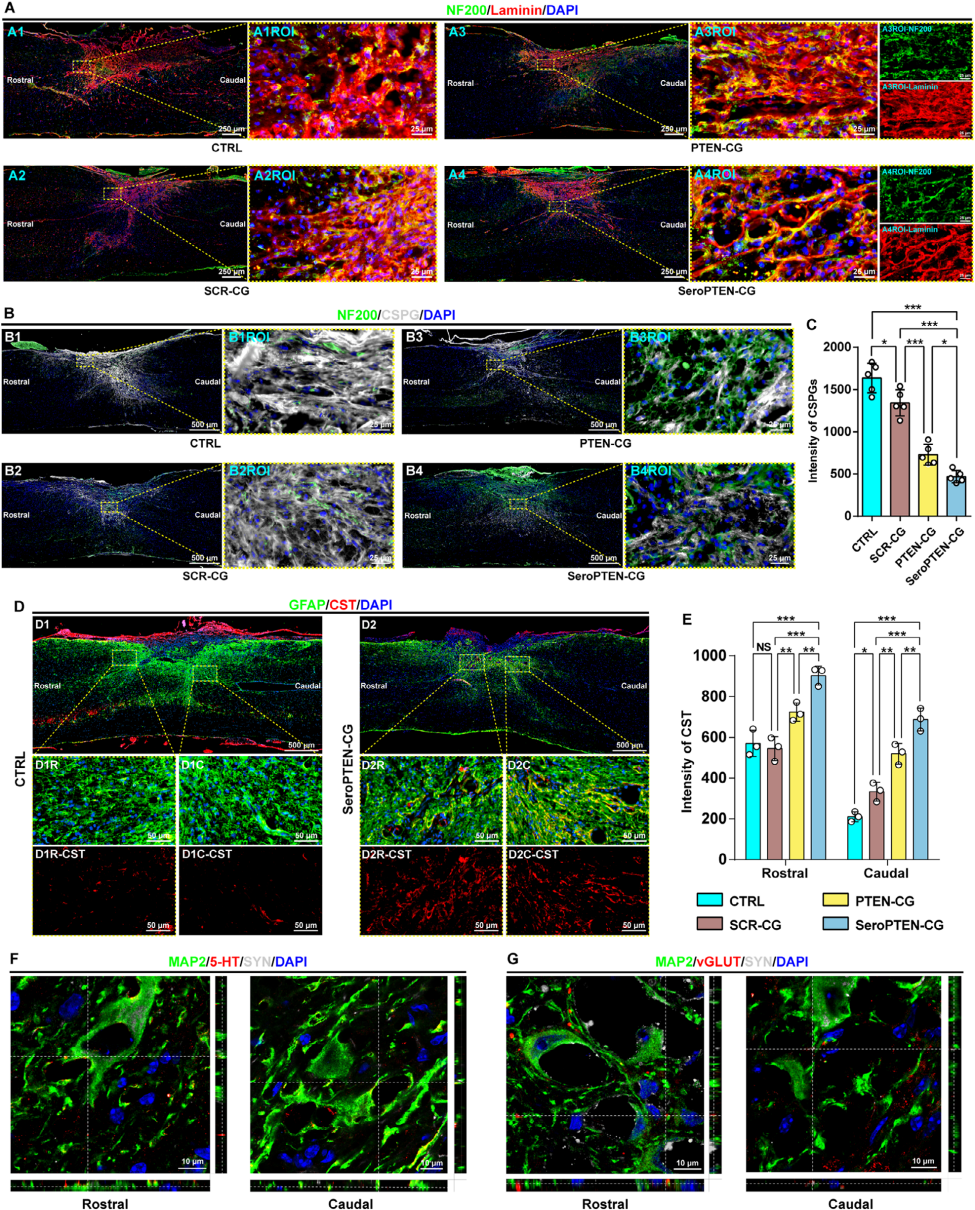
SeroPTEN‐CG remodels ECM and promotes specific circuit integration after SCI. (A) The axon‐ECM interface is shown by NF200 (green) with Ln (red). Compared with CTRL and SCR‐CG, PTEN‐CG and, especially, SeroPTEN‐CG reduce Ln burden and realign Ln fibers along the rostral‐caudal axis, and the regenerated NF200⁺ axons course along remodeled Ln (right panels A1ROI‐A4ROI). (B) Glial scar inhibition is evaluated by NF200 (green) and CSPG (gray) in vivo. Robust CSPG deposition in CTRL and SCR‐CG groups is reduced by PTEN‐CG and most prominently by SeroPTEN‐CG, and axons traverse CSPG‐rich regions in the SeroPTEN‐CG group (right panels B1ROI‐B4ROI). (C) Quantification of CSPG intensity across groups (n = 5 per group). (D) Representative spinal sections from CTRL and SeroPTEN‐CG groups show astroglial network (GFAP, green) and CST (AAV9‐hSyn‐mCherry, red). Insets (D1R, D1C, D2R, and D2C) display rostral and caudal ROIs, and the corresponding single‐channel CST images are shown below (D1R‐CST, D1C‐CST, D2R‐CST, and D2C‐CST). (E) Quantification of CST signal intensity at rostral and caudal lesion sites across groups (n = 3 per group). (F) High‐magnification images with serotonergic terminals (5‐HT, red), neurons (MAP2, green), and synaptic terminals (SYN, gray) at rostral and caudal lesion sites in SeroPTEN‐CG group. Orthogonal views (right and bottom strips) highlight colocalization of 5‐HT, MAP2, and SYN. (G) High‐magnification images with glutamatergic presynaptic terminals (vGLUT, red), neurons (MAP2, green), and synaptic terminals (SYN, gray) at rostral and caudal lesion sites in SeroPTEN‐CG group. Orthogonal views (right and bottom strips) highlight colocalization of vGLUT, MAP2, and SYN. Scale bar, as indicated. Quantitative data in (C, E) are presented as mean ± SEM. Statistical significance is assessed using one‐way ANOVA with Tukey's post hoc test for multiple comparisons. ^*^ *p* < 0.05, ^**^ *p* < 0.01, ^***^ *p* < 0.001; NS, not significant. Abbreviations: ECM, extracellular matrix; Ln, laminin; CSPG, chondroitin sulfate proteoglycan; NF200, neurofilament 200; GFAP, glial fibrillary acidic protein; CST, corticospinal tract; MAP2, microtubule‐associated protein 2; SYN, synaptophysin; vGLUT, vesicular glutamate transporter; 5‐HT, 5‐hydroxytryptamine; ROI, region of interest.

In addition to neuronal loss, secondary inflammatory cascades critically drive fibrotic scar formation through excessive synthesis and disorganized deposition of laminin (Ln), a representative component of fibrotic ECM [78]. Consistent with previous reports, the CTRL group exhibited prominent fibrotic scars characterized by dense Ln accumulation, however, PTEN‐CG and especially SeroPTEN‐CG treatments markedly reduced Ln deposition and promoted longitudinal realignment of Ln fibers along the rostral‐caudal axis (Figure 5A). Additionally, Ln fibers encapsulated NF200^+^ axons and obstructed their elongation in CTRL and SCR‐CG groups, whereas SeroPTEN‐CG treatment facilitated significant regeneration of thick and continuous axons aligned with remodeled Ln fibers (Figure 5A), indicating that ECM remodeling induced by SeroPTEN‐CG creates a more permissive microenvironment and substrates for axonal elongation. Simultaneously, glial scar was assessed by examining CSPG, a primary inhibitory component of glial scar restricting axonal growth [79, 80]. Extensive CSPG deposition occurred after SCI in CTRL group, but was significantly reduced by PTEN‐CG and most prominently by SeroPTEN‐CG treatment (Figure 5B,C), and regenerating axons in the SeroPTEN‐CG group traversed CSPG‐rich regions, a phenomenon largely absent in CTRL and SCR‐CG groups (Figure 5B).

Over the past decades, substantial advances in SCI therapy have been achieved through reconnection of interrupted tracts to restore commands between projection neurons and their downstream targets. However, considering the architectural complexity and functional specificity of spinal circuits, emphasis has shifted from mere structural reconnection toward specific spinal circuits integration [81, 82]. Accordingly, we directed attention to reconstitute relay networks with defined synaptic specificity at the lesion site.

After SeroPTEN‐CG treatment, the corticospinal tract (CST; labeled with AAV9‐hSyn‐mCherry) exhibited substantial elongation at both rostral and caudal lesion sites, suggesting more extensive connections and efficient spinal circuit reconstruction compared with other groups (Figure 5D,E; Figure S17). This structural reconstruction is consistent with the engagement of the neuron‐intrinsic PI3K‐Akt/mTOR signaling pathway, which is known to unlock adult CST axonal regeneration and collateral sprouting [10]. Notably, serotonergic axons formed synaptic contacts with MAP2^+^ neurons at lesion sites (Figure 5F), which can be attributed to the dual‐functional design of SeroPTEN‐CG. On the one hand, 5‐HTP is metabolized to enhance local 5‐HT biosynthesis and release, thereby inducing widespread activation of 5‐HTRs across interneurons and descending tracts to improve synaptic excitability. On the other hand, targeted delivery of PTEN siRNA toward the serotonergic system collectively promotes axonal elongation and circuit reconstruction for the formation of functional synaptic networks. To further validate the functional relay integration, we investigated descending glutamatergic innervation. The excitatory synaptic marker vesicular glutamate transporter (vGLUT) prominently colocalized with MAP2^+^ neurons within the lesion (Figure 5G), indicating incorporation of glutamatergic transmission into the restructured synaptic networks. The synchronized spatial colocalization of 5‐HT, vGLUT, and SYN revealed coordinated reconstruction of serotonergic and glutamatergic systems, indicating the formation of multisynaptic relay networks. Considering the complementary function of serotonergic modulation in locomotor rhythm and glutamatergic modulation in locomotor excitation, their coordinated integration provides structural foundations for engaging CPGs and descending tracts during recovery [83, 84].

Regarding the autonomic nervous system, tyrosine hydroxylase‐positive (TH^+^) axons, essential for autonomic modulation of bladder reflexes [85, 86], established synaptic contacts after SeroPTEN‐CG treatment (Figure S18A). Correspondingly, PTEN‐based Chemogene therapies, especially SeroPTEN‐CG, significantly alleviated bladder dysfunction, as evidenced by improved bladder morphology and reduced bladder volumes (Figure S18B), indicating restoration of reflexive micturition.

**FIGURE 6:**
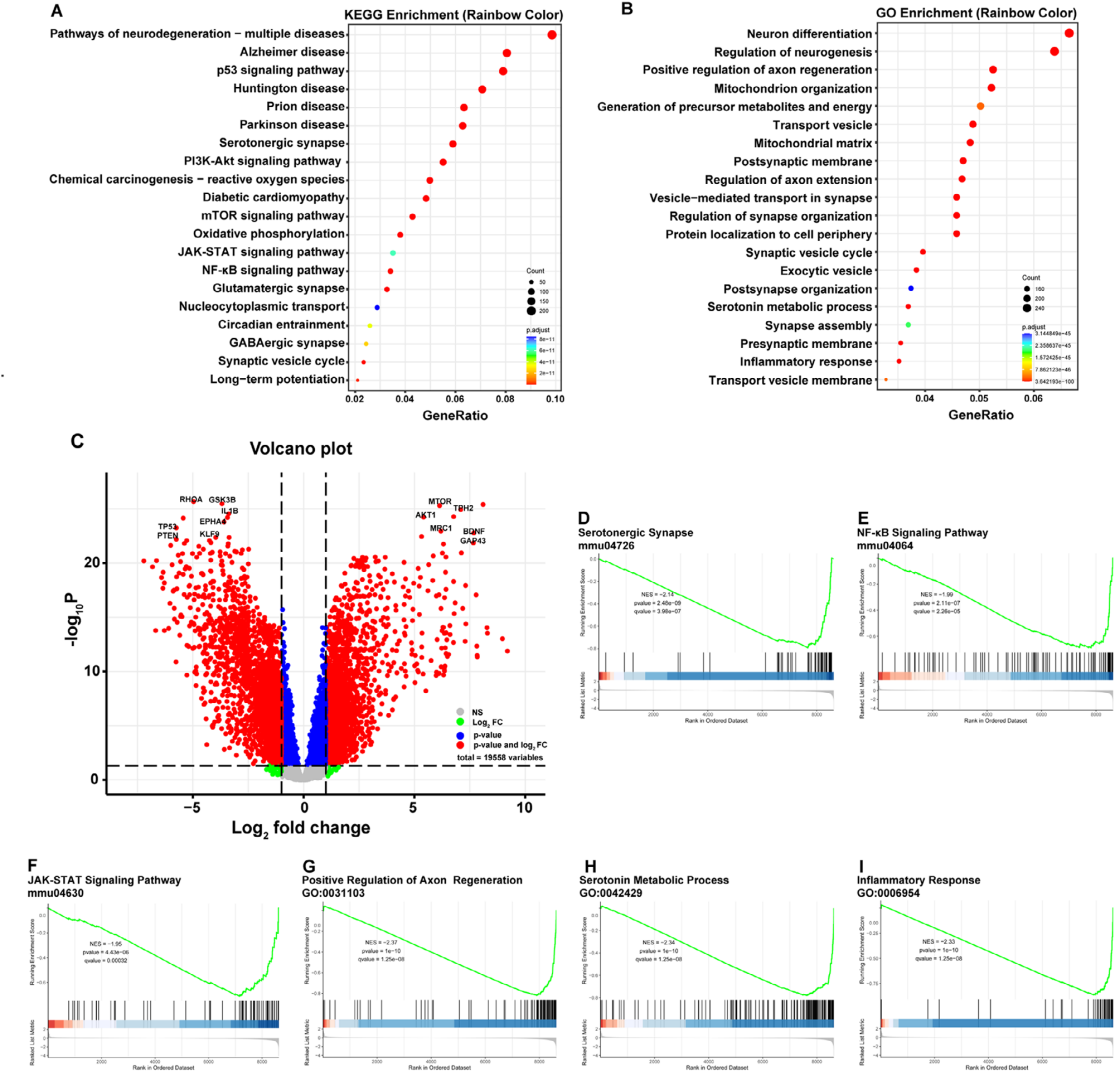
Proteomic analysis after SeroPTEN‐CG treatment. (A, B). Proteomics pathway enrichment after SeroPTEN‐CG: KEGG enrichment (A) and GO enrichment (B). (C). Volcano plot of DEPs with representative proteins annotated. (D‐F). GSEA (KEGG) demonstrates enrichment of serotonergic synapse (D), NF‐κB signaling pathway(E), and JAK‐STAT signaling pathway (F). (G‐I). GSEA (GO) demonstrates enrichment of positive regulation of axon regeneration (G), serotonin metabolic process (H), and inflammatory response (I). Abbreviations: DEPs, differentially expressed proteins; KEGG, Kyoto Encyclopedia of Genes and Genomes; GO, Gene Ontology; GSEA, Gene Set Enrichment Analysis.

Collectively, these results confirm that SeroPTEN‐CG facilitates structural and functional restoration of locomotor and autonomic spinal circuits. To elucidate underlying mechanisms, we performed proteomic analysis to delineate regulatory networks driving repair. KEGG enrichment identified the top 20 enriched pathways, including neurodegeneration‐multiple diseases, serotonergic synapse, p53 signaling pathway, PI3K‐Akt signaling pathway, mTOR signaling pathway, and glutamatergic synapse, all of which are implicated in pathophysiology of SCI therapy (Figure 6A). GO enrichment implicated processes related to neurorepair and inflammation, including neuron differentiation, regulation of neurogenesis, and cytokine‐mediated signaling (Figure 6B). The volcano plot was generated to visualize protein expression changes, with representative differentially expressed proteins (DEPs) highlighted (Figure 6C). GSEA was also applied to validate and complement the above results. Among the top enriched KEGG pathways were serotonergic synapse, nuclear factor κB (NF‐κB) signaling pathway, and Janus kinase‐signal transducer and activator of transcription (JAK‐STAT) signaling pathway (Figure 6D–F), while GO‐based GSEA revealed enrichment of positive regulation of axon regeneration, serotonin metabolic process, and inflammatory response (Figure 6G‐I). Notably, GSEA confirmed significant enrichment of both the serotonergic synapse and serotonin metabolic process, indicating strengthened serotonergic signaling at the lesion site, which is consistent with the design rationale of SeroPTEN‐CG to enhance serotonergic neuromodulation for excitability rescue. Taken together, the proteomic data reveal coordinated remodeling of neurorepair and inflammatory pathways with selective reinforcement of the serotonergic system following SeroPTEN‐CG treatment.

Collectively, ECM remodeling and specific synaptogenesis across the lesion converge on SeroPTEN‐CG as a single integrated system that synchronizes neuron‐intrinsic growth programs with neuron‐extrinsic neuroimmune optimization to reintegrate spared circuits. This unified strategy restores functional spinal networks after SCI and has potential to generalize to other CNS injuries and to inform translational strategies.

### 2.6 Functional Recovery and Spinal Circuits Reactivation

**FIGURE 7:**
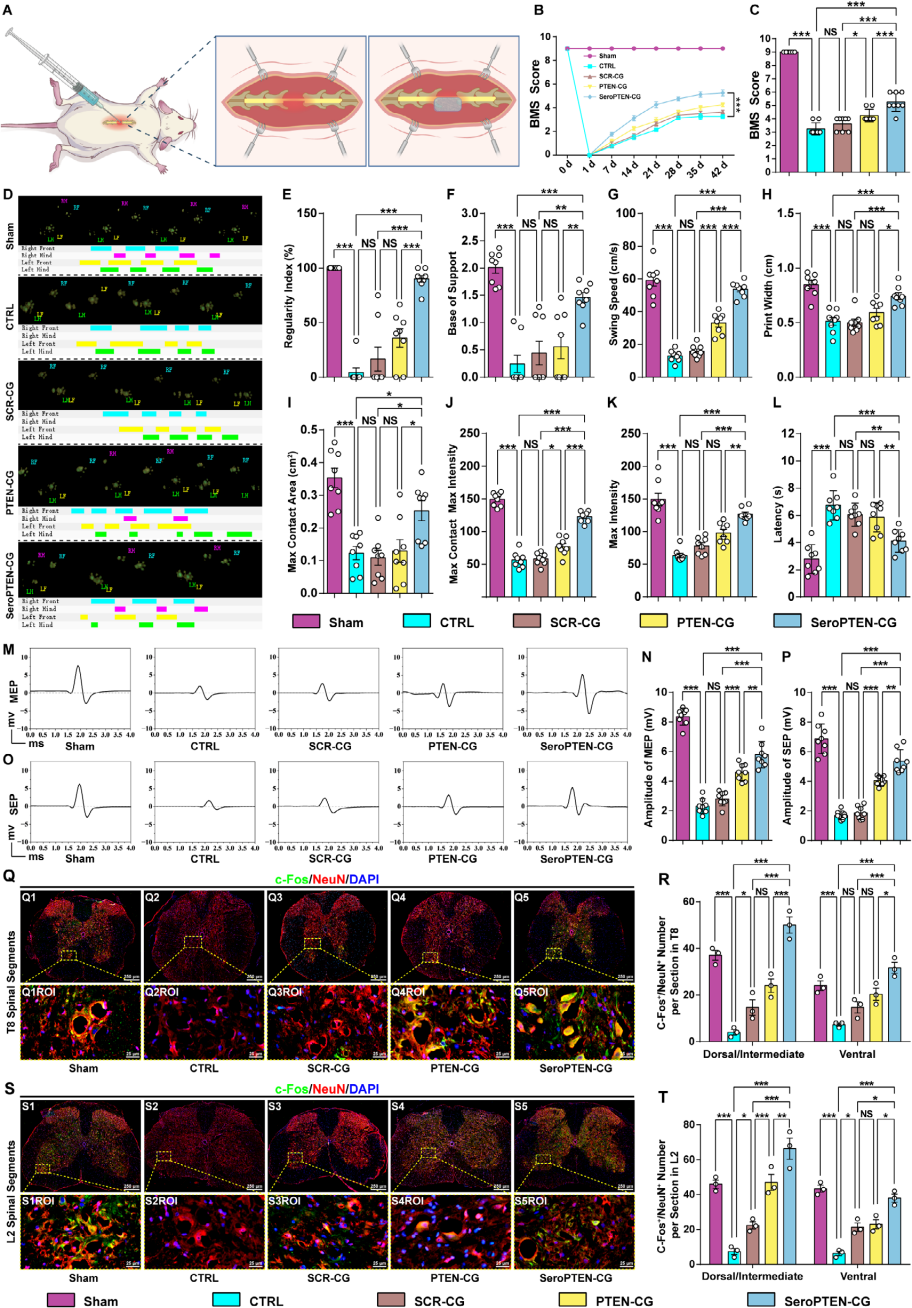
SeroPTEN‐CG improves locomotor and sensory recovery after SCI. (A). The experimental scheme of right T10 spinal cord hemisection and in situ administration of SeroPTEN‐CG within the lesion site. (B). Time course of BMS scores for Sham, CTRL, SCR‐CG, PTEN‐CG, and SeroPTEN‐CG groups over 6 weeks (n = 8 per group). (C). Group comparison of BMS scores at 6 weeks post‐injury. (n = 8 per group). (D). Representative CatWalk XT step‐sequence maps illustrate gait patterns in each group. (E‐K). Quantitative gait parameters derived from CatWalk analysis, including regularity index (E), base of support (F), swing speed (G), print width (H), maximum contact area (I), max contact max intensity (J), and max intensity (K) (n = 8 per group). (L). Plantar hot‐plate assay showing paw‐withdrawal latency in each group (n = 8 per group). (M, N). MEP recorded from hindlimb muscles: representative waveforms (M) and quantified peak amplitudes (N) (n = 8 per group). (O, P). SEP recorded from the cortex: representative waveforms (O) and quantified peak amplitudes (P) (n = 8 per group). (Q, R). Neuronal activation in thoracic segment T8, with immunofluorescence staining for c‐Fos (green) and NeuN (red), lower panels (Q1ROI‐Q5ROI) show higher magnification views of the boxed regions, and c‐Fos⁺/NeuN⁺ neurons are quantified in dorsal/intermediate and ventral regions (R) (n = 3 per group). (S, T). Neuronal activation in lumbar segment L2 with c‐Fos (green) and NeuN (red) staining, lower panels (S1ROI‐S5ROI) show higher‐magnification views of the boxed regions, and c‐Fos⁺/NeuN⁺ neurons are quantified in dorsal/intermediate and ventral regions (T) (n = 3 per group). Scale bar, as indicated. Quantitative data in (B, C, E‐L, N, P, R, T) are presented as mean ± SEM. Statistical significance for the longitudinal BMS data in (B) is assessed using two‐way repeated‐measures ANOVA with Tukey's multiple comparisons test, whereas single time‐point comparisons in (C, E‐L, N, P, R, T) are assessed using one‐way ANOVA with Tukey's post hoc test for multiple comparisons. ^*^ *p* < 0.05, ^**^ *p* < 0.01, ^***^ *p* < 0.001; NS, not significant. Abbreviations: BMS, Basso Mouse Scale; MEP, motor‐evoked potential; SEP, sensory‐evoked potential; ROI, region of interest.

The therapeutic efficacy of SeroPTEN‐CG in facilitating locomotor and sensory recovery after SCI was evaluated using Basso Mouse Scale (BMS) scoring, gait analysis, plantar hot‐plate tests, and electrophysiological assessments. All mice exhibited normal right hindlimb locomotion with a BMS score of 9, and complete paralysis (BMS = 0) was recorded immediately after right spinal cord hemisection (Figure 7A,B). Over the subsequent 6 weeks, mice in the CTRL group displayed modest spontaneous recovery with a BMS of 3.25 ± 0.16 (mean ± SEM), whereas SCR‐CG mice achieved a slight additional improvement toward more frequent plantar stepping (3.63 ± 0.18). However, both groups remained significantly inferior to the PTEN‐CG group (4.25 ± 0.25). By contrast, SeroPTEN‐CG treatment drove progressive improvement from week 1 and achieved a mean BMS of 5.25 by week 6, characterized by plantar stepping, full hindlimb weight support, efficient lift‐off, and partial forelimb‐hindlimb coordination (Figure 7B). Statistical analysis further revealed that around 40% of mice achieved a BMS of 6, while around 90% scored at least 5 by 6 weeks, reflecting the substantial therapeutic efficacy of SeroPTEN‐CG (Figure 7C).

To complement behavioral scoring with kinematic evidence, gait parameters were analyzed using CatWalk XT system. Immediately after hemisection, the right hindlimb left no paw prints and the gait parameters were near zero. Even at 6 weeks, both CTRL and SCR‐CG groups displayed only modest improvement, with irregular plantar placement and infrequent right hindlimb stepping (Figure 7D). Notably, mice in SeroPTEN‐CG group exhibited a partially coordinated gait, with a regularity index exceeding 80% (Figure 7E), suggesting reactivation and reconstruction of spared spinal circuits. Moreover, significant improvements in base of support, swing speed, and print width supported the restoration of neuromuscular coordination and postural stability after SeroPTEN‐CG treatment (Figure 7F‐H), and the concurrent improvements in maximum contact area, max contact max intensity, and max intensity (Figure 7I‐K) further indicated improved muscular engagement, neuromotor control, and limb‐ground interaction [87, 88, 89]. By comparison, these gait parameters remained deficient in CTRL and SCR‐CG groups, and exhibited only limited improvement in PTEN‐CG group (Figure 7E‐K). To extend evaluation beyond motor recovery, sensory function was evaluated using plantar hot‐plate assay. Mice treated with SeroPTEN‐CG exhibited the most pronounced sensory restoration, with the shortest withdrawal latency of approximately 4.1 seconds (s) compared to PTEN‐CG (∼5.8 s), SCR‐CG (∼6.0 s), and CTRL (∼6.7 s) groups (Figure 7L). Electrophysiology corroborated the above results, as the SeroPTEN‐CG group exhibited significant improvements in both motor‐evoked potential (MEP) and sensory‐evoked potential (SEP) amplitudes without electrophysiological signatures of hyperreflexia or hyperexcitability (Figure 7M‐P), further confirming enhanced conduction along descending motor tracts and reactivation of afferent sensory tracts in the absence of autonomic destabilization or sensory allodynia.

In light of the improvements in behavioral and electrophysiological outcomes, we asked whether spinal interneurons exhibit region‐specific activation across distinct spinal segments to elucidate underlying cellular mechanisms after SeroPTEN‐CG treatment [90]. After 1 h of treadmill walking to activate potentially excitable neurons, spinal cords were collected for immunofluorescence with c‐Fos, an immediate‐early marker of neuronal activation, and NeuN to specifically label activated neurons [91]. The quantitative analysis focused on both dorsal/intermediate and ventral regions within thoracic (T8) and lumbar (L2) segments, and the representative images of c‐Fos/NeuN double‐positive (c‐Fos^+^/NeuN^+^) neurons are illustrated. In the sham group, c‐Fos^+^/NeuN^+^ neurons were uniformly distributed across T8 and L2, whereas CTRL and SCR‐CG groups showed minimal activation at both segments (Figure 7Q,S). By contrast, SeroPTEN‐CG treatment induced a substantial increase in c‐Fos⁺/NeuN⁺ interneurons in the dorsal/intermediate and ventral regions of T8, together with a significant increase in the L2 segment (Figure 7R,T). Given the critical role of ventral regions in initiating and maintaining rhythmic movements, this ventral enrichment indicates descending commands reaching segments caudal to the lesion and reactivation of previously dormant spinal circuits [63, 92], consistent with the design rationale of SeroPTEN‐CG to integrate 5‐HT‐mediated excitability restoration with PTEN‐targeted neuron‐intrinsic growth programs to reestablish transmission of descending commands.

Multiple lines of evidence align with the mechanistic framework. CST elongation, together with 5‐HT and vGLUT synaptic labeling on MAP2⁺ neurons, indicates that relay synapses are assembled and that locomotor rhythm modulation and excitatory drive are integrated to support locomotor recovery. Consistently, improved MEP and SEP amplitudes demonstrate functional reconnection beyond structural linkage. The preferential activation of ventral c‐Fos⁺/NeuN⁺ interneurons indicates recruitment of ventral locomotor networks, consistent with restored descending input to spared circuits. Behaviorally, higher BMS scores and improved CatWalk metrics confirm recovery of coordinated stepping and controlled movement, in line with serotonergic support for rhythm and glutamatergic support for execution. Collectively, effective recovery requires excitability restoration and circuit reconstruction to be engaged together, and SeroPTEN‐CG provides an integrated pharmacologic strategy to coordinate both requirements at the lesion site.

### 2.7 In Vivo Biosafety Assessment

In vivo biosafety was evaluated by serum chemistry and histopathology. Across all groups, alanine aminotransferase (ALT), aspartate aminotransferase (AST), blood urea nitrogen (BUN), and creatinine (CREA) remained comparable and exhibited no significant differences (Figure S19), suggesting that SeroPTEN‐CG did not induce overt damage to hepatic and renal function. Consistently, H&E staining of the heart, liver, spleen, lung, and kidney revealed preserved tissue architecture without necrosis or diffuse inflammatory infiltration compared to those of the sham group (Figure S20). Together, these results indicate favorable biocompatibility of SeroPTEN‐CG under the present dosing regimen and support its suitability for SCI therapy.

## 3 Conclusion

In summary, we present SeroPTEN‐CG, modular DNA/RNA heteroduplex hydrogels that synergistically tackle two central barriers to SCI therapy: dormant neuron reactivation and spinal circuit reconstruction. Through an in situ hydrogel‐to‐nanogel transition and decoupled controllable release of 5‐HTP and PTEN siRNA, SeroPTEN‐CG restores neuronal excitability and promotes axonal elongation and synaptic reorganization, facilitating structural and functional reintegration of spared circuits. In addition, an “adaptive salvage design” converts inevitable microglial phagocytosis into neuroimmune modulation, creating a microenvironment conducive to these processes. Taken together, this work validates the feasibility of 5‐HT‐mediated functional reactivation with PTEN‐targeted structural reconstruction, and provides generalizable design principles for synergistically integrating small‐molecule pharmacology and oligonucleotide therapeutics in SCI. Importantly, the architecture of this DNA/RNA heteroduplex hydrogel is modular, swapping the targeting ligand, small‐molecule payload, or siRNA sequence readily retargets the platform to other CNS circuits and gene targets. The respective dosage and relative proportions of 5‐HTP and PTEN siRNA used here serve as a proof‐of‐concept and can be systematically optimized in subsequent studies to better align excitability support with structural reconstruction and, potentially, further improve outcomes. Future efforts will focus on dose mapping within the narrow serotonergic therapeutic window, scalable manufacturing, and expansion to additional targets to unlock greater clinical potential across CNS disorders.

## 4 Experimental Section

### 4.1 Synthesis and Characterization of SeroPTEN‐CG

#### 4.1.1 Synthesis of Compound 1

Boc‐5‐hydroxy‐DL‐tryptophan (4.00 g, 12.4 mmol) was dissolved in a mixture of tetrahydrofuran (THF, 40.0 mL) and dichloromethane (DCM, 40.0 mL). Subsequently, tert‐butyl 2,2,2‐trichloroacetimidate was added dropwise, and the resulting mixture was stirred at 25°C for 16 h. After the reaction was completed, the mixture was diluted with water (100 mL) and then extracted three times with DCM (50.0 mL each). The combined organic extracts were washed twice with brine (50.0 mL each), dried over anhydrous sodium sulfate (Na_2_SO_4_), filtered, and concentrated under reduced pressure to give a crude residue, which was then purified by silica gel chromatography (eluting with a gradient of petroleum ether (PE): ethyl acetate (EtOAc) from 5:1 to 2:1) to yield Compound 1 as a yellow oil (1.70 g, 4.52 mmol, 36.1% yield).

#### 4.1.2 Synthesis of Compound 2

Compound 1 (1.70 g, 4.52 mmol) was dissolved in acetonitrile (ACN, 10.0 mL), followed by the sequential addition of N,N‐diisopropylethylamine (DIPEA, 1.17 g, 9.03 mmol, 1.57 mL) and 2‐bromoacetic anhydride (1.17 g, 4.52 mmol). After stirring at 25°C for 3 h, the reaction was diluted with water (30 mL) and extracted three times with DCM (20.0 mL each). The organic extracts were then washed twice with brine (20.0 mL each), dried over anhydrous Na_2_SO_4_, filtered, and then concentrated under reduced pressure. Subsequently, the reaction mixture was purified by silica gel chromatography (eluting with a gradient of PE: EtOAc from 5:1 to 2:1), yielding Compound 2 as a yellow oil (1.20 g, 2.41 mmol, 53.4% yield).

#### 4.1.3 Synthesis of Compound 3

Compound 2 (800 mg, 1.61 mmol) was dissolved in DCM (5.00 mL), and trifluoroacetic acid (TFA, 1.47 g, 12.8 mmol, 955 µL) was then added with stirring. After stirring at 25°C for 30 min, the reaction mixture was concentrated under reduced pressure to obtain the crude residue, which was then purified by high‐performance liquid chromatography (HPLC). Compound 3 was obtained as a light‐yellow solid (458 mg, 1.16 mmol, 72.3% yield, purity: 96.0%).

#### 4.1.4 Synthesis of 5‐HTP‐DNA Conjugates

The phosphorothioate (PS) ‐ bearing DNA strands (100 µM, Ya‐2PS, Yb‐2PS, Yc‐2PS) and Compound 3 (6 mM) were respectively dissolved in dimethyl sulfoxide (DMSO) and reacted for 30 min with gentle shaking at 50°C. After the ethanol precipitation and vacuum evaporation, the 5‐HTP‐modified DNA (5‐HTP‐Ya‐2PS, 5‐HTP‐Yb‐2PS, 5‐HTP‐Yc‐2PS) were obtained and stored at −20°C for further use and analysis. The molecular weight analysis of representative 5‐HTP‐modified DNA was performed by liquid chromatography‐mass spectrometry (LC‐MS) (Thermo Fisher Scientific, USA), and the 15% denaturing PAGE was employed to analyze the 5‐HTP‐modified DNA in 1× Tris‐borate‐EDTA (TBE) buffer (89 mM Tris, 89 mM boric acid, and 2 mM EDTA), after running for about 1 h, the gels were stained in ethidium bromide solution for approximately 1 min and subsequently imaged using Bio‐Rad imaging system.

#### 4.1.5 Synthesis of 5‐HTP‐Modified Y‐Motifs and the SeroPTEN‐CG

To construct the 5‐HTP‐modified Y‐motifs, three kinds of 5‐HTP‐DNA conjugates (5‐HTP‐Ya‐2PS, 5‐HTP‐Yb‐2PS, 5‐HTP‐Yc‐2PS) with equimolar quantities were mixed in 1×TAE/Mg^2+^ buffer (40.0 mM Tris, 2.0 mM EDTA·Na_2_·H_2_O, 20.0 mM acetic acid, 12.5 mM (CH_3_COO)_2_Mg·4H_2_O), then the mixture was heated up to 90°C for 5 min and swiftly cooled down to 4°C to facilitate the formation of 5‐HTP‐modified Y‐motifs. Subsequently, SeroPTEN‐CG was assembled by mixing the 5‐HTP‐modified Y‐motifs and the PTEN siRNA‐linkers at a Y‐motif: linker molar ratio of 1:1.5. After which, the representative SeroPTEN‐CG was characterized in 1× TAE/Mg^2+^ running buffer using 10% native PAGE, and the gels were stained in ethidium bromide solution and imaged after running for about 1 h. FAM‐ and Cy5.5‐modified constructs were prepared under the same conditions by replacing linker‐2 with FAM‐linker‐2 and Cy5.5‐linker‐2, respectively. In practical use, the SeroPTEN‐CG liquefies on heating to 90°C and spontaneously re‐gelates within approximately 1 min after returning to room temperature.

#### 4.1.6 In Vitro Degradation Assessment

In accordance with the instructions of manufacturers, SeroPTEN‐CG was incubated at 37°C for 12 h with gradient concentrations of DNase I (5, 10, 25, 50 U/mL), then the obtained samples were centrifuged at 4000 rpm for 5 min to remove residual undegraded SeroPTEN‐CG, and the supernatants were isolated and the size distribution of disassembled particles was measured using dynamic light scattering (DLS) (Zetasizer Nano S, Malvern Instruments, UK).

#### 4.1.7 RNase H‐Mediated siRNA Release Kinetics

To characterize the release of PTEN siRNA, the SeroPTEN‐CG was incubated with gradient concentrations of RNase H (0, 10, 20, 50, 100 U/mL) at 37°C for 1 h. Subsequently, 10% native PAGE was employed to characterize the release of PTEN siRNA from post‐digestion samples, and the resultant gels were imaged using a Bio‐Rad imaging system.

#### 4.1.8 5‐HTP Release Kinetics

Cumulative release of 5‐HTP under different conditions was quantified by high‐performance liquid chromatography (HPLC). SeroPTEN‐CG samples were incubated in four media: phosphate buffer (pH 7.4), acetate buffer (pH 5.0), phosphate buffer (pH 7.4) containing esterase (17 U/mL), and acetate buffer (pH 5.0) containing esterase (17 U/mL). Incubations were maintained at 37°C with shaking at 100 rpm. At predetermined intervals (0, 2.5, 5, 10, 20, 30, 48, and 72 h), samples were collected and analyzed by HPLC to quantify 5‐HTP, from which cumulative release profiles were derived.

### 4.2 In Vitro Cellular Experiments

#### 4.2.1 Isolation and Cultivation of Primary Neurons

Primary neurons were obtained from the embryonic cortices of C57BL/6 mice (GemPharmatech, China), and all animal experiments were performed in accordance with ethical guidelines approved by the Ethics Committee of Shandong University. Briefly, pregnant mice at gestational day 16 were sacrificed and sterilized with 75% pre‐cooled ethanol, then the cortical regions were dissected under a stereomicroscope (Chongqing Optec Instrument Co., Ltd., China), minced into approximately 1 mm^3^ fragments, and enzymatically digested for 20 min at 37°C in a solution containing 0.2% (w/v) papain (Worthington Biochem, USA) and 0.004% (w/v) DNase (Sigma‐Aldrich, USA), accompanied by gentle pipetting (8‐10 strokes every 5 min). After the digestion, the cell suspension was centrifuged at 200 × g for 5 min, and cell clumps were carefully aspirated off and filtered through a 40 µm cell strainer (SPL Life Sciences, ROK). The harvested cells were resuspended in Dulbecco's Modified Eagle Medium (Invitrogen, USA) containing 10% fetal bovine serum (Cellmax, China) and 1% penicillin‐streptomycin (Gibco, USA) before being seeded onto wells coated with poly‐D‐lysine (Sigma‐Aldrich, USA). Four hours later, the medium was replaced with neuronal medium (Neurobasal, 2% B27 supplement, Invitrogen; 1 mM L‐glutamine, Invitrogen; and 1% penicillin‐streptomycin, Invitrogen), with half of the medium refreshed every 3 days.

#### 4.2.2 Protein Docking and Visualization

Receptor protein structures were initially processed and optimized using PyMOL, involving the elimination of heteroatoms, structural repair of incomplete residues, and refinement for accurate molecular docking studies. Small‐molecule ligands were generated as optimized 3D structures based on their respective 2D chemical formulas. Both receptor proteins and ligands were individually prepared for docking using AutoDock Tools (ADT) [93]. Molecular docking simulations were performed utilizing AutoDock Vina software [94, 95] to comprehensively evaluate global binding interactions between ligands and their corresponding receptor proteins.

#### 4.2.3 In Vitro Targeting Capability and Cellular Internalization of SeroPTEN‐CG

For in vitro targeting capability analysis of SeroPTEN‐CG, SERT‑HEK293 cells (HEK293 cells engineered to express the serotonin transporter, SERT) or 5‐HT_2A_R‑HEK293 cells (HEK293 cells engineered to express 5‐HT_2A_R) were seeded into Transwell plate (NEST, China) at a density of 4 × 10^4^ cells per well and incubated overnight at 37°C for cell adhesion. Subsequently, the culture medium containing SeroPTEN‐CG was introduced into the upper chamber and incubated with SERT‑HEK293 cells or 5‐HT_2A_R‑HEK293 cells for an additional 12 h. After incubation, cells were fixed with 4% paraformaldehyde for 10 min, followed by immunofluorescence staining. The targeting capability was visualized using confocal laser scanning microscopy.

For the evaluation of cellular internalization, primary neurons were seeded into 24‐well plates at a density of 4 × 10^4^ cells per well and allowed to adhere overnight at 37°C, and then treated with culture medium containing FAM‐labeled SeroPTEN‐CG nanogels (FAM concentration equivalent to 2 µM). After incubation for 0, 1, 2, 4, and 6 h, the cells were detached using 0.25% trypsin solution (Gibco, USA), collected, and analyzed by FCM (LSRFortessa, Becton Dickinson Immunocytometry Systems, USA) for uptake determination.

#### 4.2.4 Axonal Guidance Spot Assays

For CSPG‐mediated axonal guidance spot assays, 20 mm glass‐bottom culture dishes (NEST, China) were coated with poly‐L‐lysine at room temperature overnight, and subsequently spotted at the center with 5 µL droplets containing chicken CSPG (Millipore, USA) mixed with Texas Red dye (Thermo Fisher Scientific, USA) to delineate the CSPG boundary. After drying the spots completely, primary neurons were seeded onto glass‐bottom cell culture dishes at a density of 8 × 10⁴ cells and incubated for 48 h with treatment with SeroPTEN‐CG, PTEN‐CG, or SCR‐CG. After the incubation, the neurons were then fixed, and immunofluorescent labeling was performed using an anti‐βIII‐tubulin antibody (Abcam, UK), followed by incubation with a fluorophore‐conjugated secondary antibody. The neuronal images were acquired utilizing an inverted confocal microscope, and the axonal lengths were analyzed by employing ImageJ image analysis software (NIH, USA).

#### 4.2.5 Axonal Outgrowth Assays

For CSPG‐mediated axonal outgrowth assays, 20 mm glass‐bottom culture dishes (NEST, China) were initially coated with poly‐L‐lysine at room temperature overnight, followed by cautious addition of CSPG solution (2.5 µg/mL) and incubation at 37°C for an additional 6 h. Subsequently, primary neurons were seeded onto the prepared glass‐bottom cell culture dishes at a density of 8 × 10⁴ cells per well, and cultured for 48 h in medium containing SeroPTEN‐CG, PTEN‐CG, or SCR‐CG. Following incubation, neurons were fixed with 4% paraformaldehyde and immunostained using an anti‐βIII‐tubulin primary antibody (Abcam, UK), followed by visualization with a suitable fluorescent secondary antibody. The images of the neurons were captured using an inverted confocal microscope, and measurements of axonal length were conducted using ImageJ software (NIH, USA).

### 4.3 In Vivo Animal Experiments

#### 4.3.1 Mice Spinal Cord Hemisection Model

A right T10 hemisection model was established in female C57BL/6 mice, which generates a highly reproducible unilateral lesion cavity with a sharply defined rim of spared spinal tissue and thereby provides an anatomically stable substrate to assess SeroPTEN‐CG mediated excitability restoration and circuit reconstruction [96, 97, 98]. The mice were maintained under standardized laboratory conditions, including a 12‐hour light/dark cycle, temperature control at 20–25°C, and humidity maintained between 40% and 60%. Before spinal cord hemisection surgery, mice were acclimated to the laboratory environment for 3 days, followed by 12 h fasting period without food and water, and then anesthetized with isoflurane (R510‐22, RWD Life Science Co., Ltd., China). The skin, fascia, and muscles were sequentially dissected to expose the vertebral lamina at thoracic level T10, after which a T10 laminectomy was performed to clearly expose the spinal cord. Subsequently, an approximately 1 mm spinal cord segment located to the right of T10 was precisely transected using a microscalpel, and the pre‐prepared SeroPTEN‐CG, PTEN‐CG, or SCR‐CG was carefully implanted within the defect site of the spinal cord after aspirating residual tissue fluid and thoroughly removing remaining tissue. After the successful spinal cord hemisection was verified by observing immediate tail spasms and right hindlimb paralysis, the muscles, fascia, and skin were then sutured in sequence. After the surgery, the bladders of the animals were manually emptied twice daily, and cefuroxime sodium (6 mg/mL dissolved in sterile saline; Hongtu, China) was administered intramuscularly to minimize the risk of urinary tract infections.

#### 4.3.2 In Vivo Targeting Capability

To investigate the targeting capability of SeroPTEN‐CG following spinal cord injury in vivo, the Cy5.5‐labeled SeroPTEN‐CG or Cy5.5‐labeled PTEN‐CG was administered directly into lesion site after spinal cord hemisection for comparative analysis. 24 h after surgery, the mice were euthanized, and systemic perfusion was performed with 50 mL of ice‐cold PBS to remove circulating fluorescent particles. Subsequently, the spinal cord tissues were harvested and fixed in 4% paraformaldehyde, cryoprotected with sucrose, embedded, and cryosectioned at a thickness of 6 µm, then the immunofluorescent staining was performed using primary antibodies against NF200 (Rabbit, 1:5000, Abcam, ab8135), MAP2 (Chicken, 1:5000, Abcam, ab5392), and 5‐HT (Goat, 1:5000, immunostar, 20079), followed by visualization with a suitable fluorescent secondary antibody. The colocalization was imaged using the whole landscape imaging system (PerkinElmer, Vectra Polaris, USA) and analyzed with ImageJ software (NIH, USA).

#### 4.3.3 Behavioral Analysis—Basso Mouse Scale (BMS)

Hindlimb locomotor recovery after spinal cord injury was assessed using the BMS. BMS scoring was conducted at baseline (pre‐injury), at days 1 and 7 post‐injury, and weekly thereafter up to 6 weeks. Mice were acclimated to a circular open‐field area (1 m diameter) enclosed by transparent walls prior to testing, allowing unobstructed observation of locomotor behavior. Locomotion tests were performed by two independent observers blinded to the experimental group assignments to minimize observational bias. Each mouse was placed individually for a 4 min observation period, during which hindlimb movements were evaluated and scored according to the BMS criteria. The BMS scores range from 0 to 9, with a score of 0 denoting no spontaneous hindlimb movement and a score of 9 corresponding to normal hindlimb locomotion.

#### 4.3.4 Behavioral Analysis—Catwalk Gait

The footprints, locomotor behavior, and physical coordination after spinal cord injury were assessed using the CatWalk XT automated gait analysis system (Noldus, the Netherlands), and all gait assessments were performed in a quiet, darkened environment to minimize external interference. Prior to testing, mice were trained to voluntarily cross the walkway at least 3 times to ensure consistent locomotor behavior and to habituate to the apparatus. During each trial, paw placement patterns of each mouse were recorded by the digital video camera, and the key spatiotemporal parameters were automatically extracted using the CatWalk XT.

#### 4.3.5 Behavioral Analysis—Sensation Assessment

Thermal sensitivity was measured at 6 weeks post‐injury to evaluate sensory recovery following spinal cord injury. Each mouse was individually placed on a heated plantar hot plate maintained at 55°C, and the latency to right hindlimb withdrawal or flicking was recorded as a quantitative indicator of thermal responsiveness. To ensure reliability, each mouse underwent 3 independent measurements with sufficient rest intervals. All procedures were conducted under controlled temperature and lighting conditions to minimize environmental variability.

#### 4.3.6 Behavioral Analysis—Electrophysiological Recording

Electrophysiological analysis of MEP was performed to assess neural recovery after spinal cord injury. In brief, mice were anesthetized with pentobarbital at a dosage of 30 mg/kg prior to testing, and the MEP signals from the hindlimb were recorded using an electrophysiological recording system (YRKJ‐G2008; Zhuhai Yiruikeji Co., Ltd., China) with a stimulation intensity of 5 mA. The stimulating electrode was accurately positioned above the cortical motor region, and the corresponding recording electrode was implanted into the opposite hindlimb muscle. SEP was recorded following stimulation of the peripheral nerve with a constant current stimulator. The amplitude of the MEP and SEP, reflecting the peak voltage of the waveform, was determined by the vertical (y‐axis) distance from the baseline to the peak of the P wave.

#### 4.3.7 Anterograde Tracing

To examine corticospinal tract (CST) regeneration after spinal cord injury, anterograde labeling was performed via intracortical injection of AAV9‐hSyn‐mCherry at 28 days post‐injury. Mice were anesthetized with isoflurane and positioned in a stereotaxic instrument. Following a midline scalp incision, the bregma was exposed, serving as the stereotaxic reference for localizing the cortical motor area. AAV9‐hSyn‐mCherry was injected into four sites with the following coordinates: anteroposterior (AP) +0.7 mm, mediolateral (ML) ±1.2 mm; AP −0.7 mm, ML ±1.2 mm; dorsoventral (DV) −0.6 mm. At each site, 100 nL of AAV9‐hSyn‐mCherry was injected using a microsyringe at a rate of 50 nL/min, and the injection needle was held in place for 10 min to prevent viral leakage. Two weeks later, animals were transcardially perfused, and the spinal cord samples were collected for fluorescent imaging under confocal microscope to evaluate CST labeling and regenerative response.

#### 4.3.8 Spinal Cord Dissection and Sectioning

Mice were anesthetized, and the thoracoabdominal cavity was exposed through a bilateral incision along the costal margins. Transcardial perfusion was initiated with ice‐cold PBS to flush out circulating blood, followed by infusion of 4% paraformaldehyde once the liver became pale and the outflow from the right atrium turned clear. After perfusion, the vertebral column was exposed by carefully removing the bilateral lamina using spring scissors, and the spinal cord was excised and fixed overnight in 4% paraformaldehyde at 4°C. Samples were then cryoprotected in a graded sucrose solution, embedded in optimal cutting temperature (OCT) compound, and sectioned at a thickness of 6 or 25 µm (for CST tracing) using a cryostat (Leica CM3050S, Germany).

#### 4.3.9 H&E Staining and Morphological Assessment

Tissue sections were stained using a standard hematoxylin and eosin (H&E) protocol for general histological assessment. The tissue sections were immersed in hematoxylin solution (Phygene, China) for 5 min to label nuclear structures, rinsed in distilled water for 10 s, and then immersed in hydrochloric acid ethanol solution for 3 s to differentiate excess stain. After an additional rinse in distilled water for 30 s, eosin solution (Phygene, China) was applied for 5 min to stain cytoplasmic components. After completion of staining, the sections were dehydrated through a graded ethanol series (80%, 90%, 95%, and 100%) and subsequently cleared in xylene for 5 min, and finally mounted with neutral resin. The histological images were captured using a NanoZoomer S60 (Hamamatsu Photonics K.K., Japan).

#### 4.3.10 Serum Biochemical Analysis

At the study endpoint, whole blood was collected into serum separator tubes, allowed to clot at room temperature for 2 h, and centrifuged at 3000 rpm for 15 min to obtain serum. Then the resulting serum was immediately analyzed for alanine aminotransferase (ALT), aspartate aminotransferase (AST), blood urea nitrogen (BUN), and creatinine (CREA) on an automated chemistry analyzer (Chemray 800, Rayto Life & Analytical Sciences, China) according to the manufacturer's instructions.

### 4.4 Western Blot Analysis

Total proteins were extracted using radioimmunoprecipitation assay (RIPA) lysis buffer (Beyotime, China) supplemented with 1% phenylmethylsulfonyl fluoride (PMSF), ethylenediaminetetraacetic acid (EDTA), and a protease inhibitor cocktail, and then homogenates were centrifuged at 12 000 × g for 20 min at 4°C. The resulting supernatants were collected, and the protein concentrations were measured using a bicinchoninic acid (BCA) kit (Beyotime, China) following the manufacturer's protocol. Equal amounts of total protein were separated by SDS‐polyacrylamide gel electrophoresis and subsequently transferred onto polyvinylidene fluoride (PVDF) membranes (Millipore, USA), which were then blocked for 1 h with Tris‐buffered saline with Tween‐20 (TBST) containing 5% bovine serum albumin (BSA, Biofroxx, Germany), followed by overnight incubation at 4°C with specific primary antibodies. After washing three times with TBST, the PVDF membranes were incubated with horseradish peroxidase (HRP)‐conjugated secondary antibodies for 1 h at room temperature. Finally, the protein bands were visualized using a chemiluminescent (ECL) reagent (Thermo Fisher Scientific, USA), and the images were captured using the chemiluminescent imaging system (Bio‐Rad, USA). The quantitative analysis of protein band intensities was performed using ImageJ software (NIH, USA), relative protein expression was normalized to GAPDH.

### 4.5 qRT‐PCR Analysis

Total RNA was extracted using TRIzol reagent (Ambion, USA) according to the manufacturer's instructions, and the purity and concentration of RNA were assessed using a NanoDrop One Spectrophotometer (Thermo Fisher Scientific, USA). Then the RNA was reverse transcribed into complementary DNA (cDNA) using the RevertAid First Strand cDNA Synthesis Kit (Thermo Fisher Scientific, USA), and the quantitative PCR was performed using PowerUp SYBR Green Master Mix (Thermo Fisher Scientific, USA) on a QuantStudio Real‐Time PCR System (Applied Biosystems, USA). Relative gene expression levels of target genes were calculated using the ΔΔCt method, with GAPDH serving as the endogenous control.

### 4.6 Bioinformatics Analysis

Raw RNA‐seq count data were analyzed using DESeq2, edgeR, and limma‐voom in R (v4.2.3). Genes with average CPM > 1 were retained. DESeq2 used a negative binomial model with median‐of‐ratios (library size); edgeR applied trimmed mean of M‐values (TMM) normalization and generalized linear model (GLM) fitting; limma used voom transformation with mean‐variance modeling. DEGs were defined as adjusted *P* < 0.05 and |log_2_FC| > 1. DESeq2 results were used for downstream analysis. The study identified significant DEPs using the UniProt database. Bioinformatics analysis followed, utilizing Proteome Discoverer 2.2 to search spectra from each run. DEPs were defined by statistical analysis as features with adjusted *P* < 0.05 and |log_2_FC| > 1. Volcano plots for both transcriptome and proteome data were generated using the EnhancedVolcano R package, highlighting DEGs/DEPs with adjusted *P* < 0.05 and |log_2_FC| > 1. Key genes of interest were manually annotated based on prior knowledge or experimental relevance. For over‐representation analysis, DEGs/DEPs (adjusted *P* < 0.05) were analyzed for GO and KEGG enrichment using clusterProfiler. Gene symbols were converted to Entrez IDs through org.Mm.eg.db, and enrichment was performed separately for GO categories (biological process, cellular component, molecular function) and KEGG pathways. Results with adjusted *P* < 0.05 were considered significant. In parallel, GSEA was conducted on preranked gene lists sorted by log_2_FC using gseKEGG in clusterProfiler. Default parameters were applied with a gene set size between 10 and 500, and adjusted *P* < 0.05 as the significance threshold. Top KEGG pathways were visualized using the GseaVis package, with annotations of normalized enrichment scores and core enriched genes. All analyses and visualizations were performed in R unless otherwise stated. Scripts are available upon request.

### 4.7 Statistical Analysis

All statistical analyses were performed using GraphPad Prism 9 (GraphPad Software, USA), and all the data were presented as mean ± standard error of the mean (SEM). Group differences at a single time point were evaluated using one‐way ANOVA with Tukey's post hoc test, whereas longitudinal data were analyzed using two‐way repeated‐measures ANOVA with Tukey's multiple comparisons test. Differences were considered statistically significant at *p* < 0.05.

## Conflicts of Interest

The authors declare no conflict of interest.

## Supporting information




**Supporting File**: adma71986‐sup‐0001‐SuppMat.docx

## Data Availability

The data that support the findings of this study are available from the corresponding author upon reasonable request.
