# Supplementary material for: Serotonergic System‐Targeted Nucleic Acid Hydrogel Coordinates Excitability Restoration and Circuit Reconstruction for Spinal Cord Injury Therapy
Source: Adv Mater. 2026 Jan 13;38(11):e21427. doi: 10.1002/adma.202521427 (PMC12921341; doi:10.1002/adma.202521427)
Supplement: Supplementary file 1 — Supporting File: adma71986‐sup‐0001‐SuppMat.docx [file ADMA-38-e21427-s001.docx]

**Serotonergic System-Targeted Nucleic Acid Hydrogel Coordinates Excitability Restoration and Circuit Reconstruction for Spinal Cord Injury Therapy**

Chunlin Li, Xiaoqing Zhao, Kai Jiang, Haixia Kang, Shuo Liu, Linlin Jiang, Baoshuai Bai, Haonan Cui, Ziyang Zhang, Menglei Dong, Ruizhi Zhang, Chenbo Zou, Shenghui Shang, Chi Zhang, Xiangchuang Fan, Lijuan Zhu, Huiquan Duan*, Chuan Zhang*, Shiqing Feng*, Hengxing Zhou*

Chunlin Li, Xiaoqing Zhao, Kai Jiang, Haixia Kang, and Shuo Liu contributed equally to this work.

Chunlin Li, Xiaoqing Zhao, Linlin Jiang, Baoshuai Bai, Ziyang Zhang, Ruizhi Zhang, Chenbo Zou, Shenghui Shang, Xiangchuang Fan, Huiquan Duan, Shiqing Feng, Hengxing Zhou
Department of Orthopaedics, Qilu Hospital of Shandong University, Shandong University Centre for Orthopaedics, Advanced Medical Research Institute, Cheeloo College of Medicine, Shandong University, Jinan, Shandong 250012, P.R. China

Chi Zhang, Shiqing Feng
Department of Orthopaedics, The Second Qilu Hospital of Shandong University, Cheeloo College of Medicine, Shandong University, Jinan, Shandong 250033, P.R. China

Haonan Cui, Menglei Dong, Chuan Zhang
School of Chemistry and Chemical Engineering, Frontiers Science Center for Transformative Molecules, Shanghai Key Laboratory for Molecular Engineering of Chiral Drugs, Shanghai Jiao Tong University, 800 Dongchuan Road, Shanghai 200240, P.R. China

Shiqing Feng
Department of Orthopaedics, Tianjin Medical University General Hospital, Tianjin Medical University, International Science and Technology Cooperation Base of Spinal Cord Injury, Tianjin Key Laboratory of Spine and Spinal Cord, Tianjin 300052, P.R. China

Kai Jiang
Department of Ophthalmology, Eye & ENT Hospital, State Key Laboratory of Brain Function and Disorders, Fudan University, Shanghai 200031, P.R. China

Lijuan Zhu
Institute of Molecular Medicine, Shanghai Jiao Tong University Affiliated Renji Hospital, Shanghai Jiao Tong University School of Medicine, Shanghai 200217, P.R. China

Haixia Kang
Department of Liver Surgery and Transplantation and Key Laboratory of Carcinogenesis and Cancer Invasion, Ministry of Education, Liver Cancer Institute, Zhongshan Hospital, Fudan University, Shanghai 200032, P.R. China

Shuo Liu
Department of Geriatric Medicine, Qilu Hospital of Shandong University, Wenhuaxi Road 107, Jinan, Shandong 250012, P.R. China

**Corresponding authors:**

Hengxing Zhou
Department of Orthopaedics, Qilu Hospital of Shandong University, Shandong University Centre for Orthopaedics, Advanced Medical Research Institute, Cheeloo College of Medicine, Shandong University, Jinan, Shandong 250012, P.R. China
E-mail: zhouhengxing@sdu.edu.cn

Shiqing Feng
Department of Orthopaedics, Qilu Hospital of Shandong University, Shandong University Centre for Orthopaedics, Advanced Medical Research Institute, Cheeloo College of Medicine, Shandong University, Jinan, Shandong 250012, P.R. China
Department of Orthopaedics, The Second Qilu Hospital of Shandong University, Cheeloo College of Medicine, Shandong University, Jinan, Shandong 250033, P.R. China
Department of Orthopaedics, Tianjin Medical University General Hospital, Tianjin Medical University, International Science and Technology Cooperation Base of Spinal Cord Injury, Tianjin Key Laboratory of Spine and Spinal Cord, Tianjin 300052, P.R. China
E-mail: shiqingfeng@sdu.edu.cn

Chuan Zhang
School of Chemistry and Chemical Engineering, Frontiers Science Center for Transformative Molecules, Shanghai Key Laboratory for Molecular Engineering of Chiral Drugs, Shanghai Jiao Tong University, 800 Dongchuan Road, Shanghai 200240, P.R. China
E-mail: chuanzhang@sjtu.edu.cn

Huiquan Duan
Department of Orthopaedics, Qilu Hospital of Shandong University, Shandong University Centre for Orthopaedics, Advanced Medical Research Institute, Cheeloo College of Medicine, Shandong University, Jinan, Shandong 250012, P.R. China
E-mail: duan_hq@tmu.edu.cn

**Keywords:**

Spinal Cord Injury; Nucleic Acid Hydrogel; Serotonergic System; Neuronal Reactivation; Circuit Reconstruction

**Abstract:**

Despite the persistence of spared spinal circuits capable of relaying commands after spinal cord injury (SCI), their contribution to recovery remains constrained by functional dormancy of spared neurons and impaired reconnection across the lesion. Serotonergic neuromodulation is pivotal for reactivating dormant neurons, however, achieving precise targeting and modulation of serotonergic system poses translational challenges. Here a DNA/RNA heteroduplex hydrogel is reported that integrates 5-hydroxytryptamine (5-HT)-mediated neuronal excitability restoration with phosphatase and tensin homolog (PTEN)-targeted spinal circuit reconstruction for SCI therapy. The 5-hydroxytryptophan (5-HTP)-derived motif, serving both as a targeting ligand and as a neuromodulator, is site-specifically grafted onto three phosphorothioate-bearing single-stranded DNA (ssDNA) strands, which self-assemble into Y-shaped motifs and are subsequently crosslinked by sticky-ended PTEN small interfering RNA (siRNA) to form the hydrogel network. After lesion-site administration, the hydrogel undergoes DNase-mediated network disassembly into nanogels that exert two complementary therapeutic actions by targeting serotonergic system: restoring excitability to reactivate dormant interneurons and reconstructing descending connectivity to reintegrate spared circuits with host spinal cord, thereby restoring sensory and locomotor functions in paralyzed mice. This strategy coordinately reinstates functional excitability and structural rebuilding by engaging multiple interlocking mechanisms, advancing a versatile paradigm for integrative therapy of central nervous system (CNS) disorders.


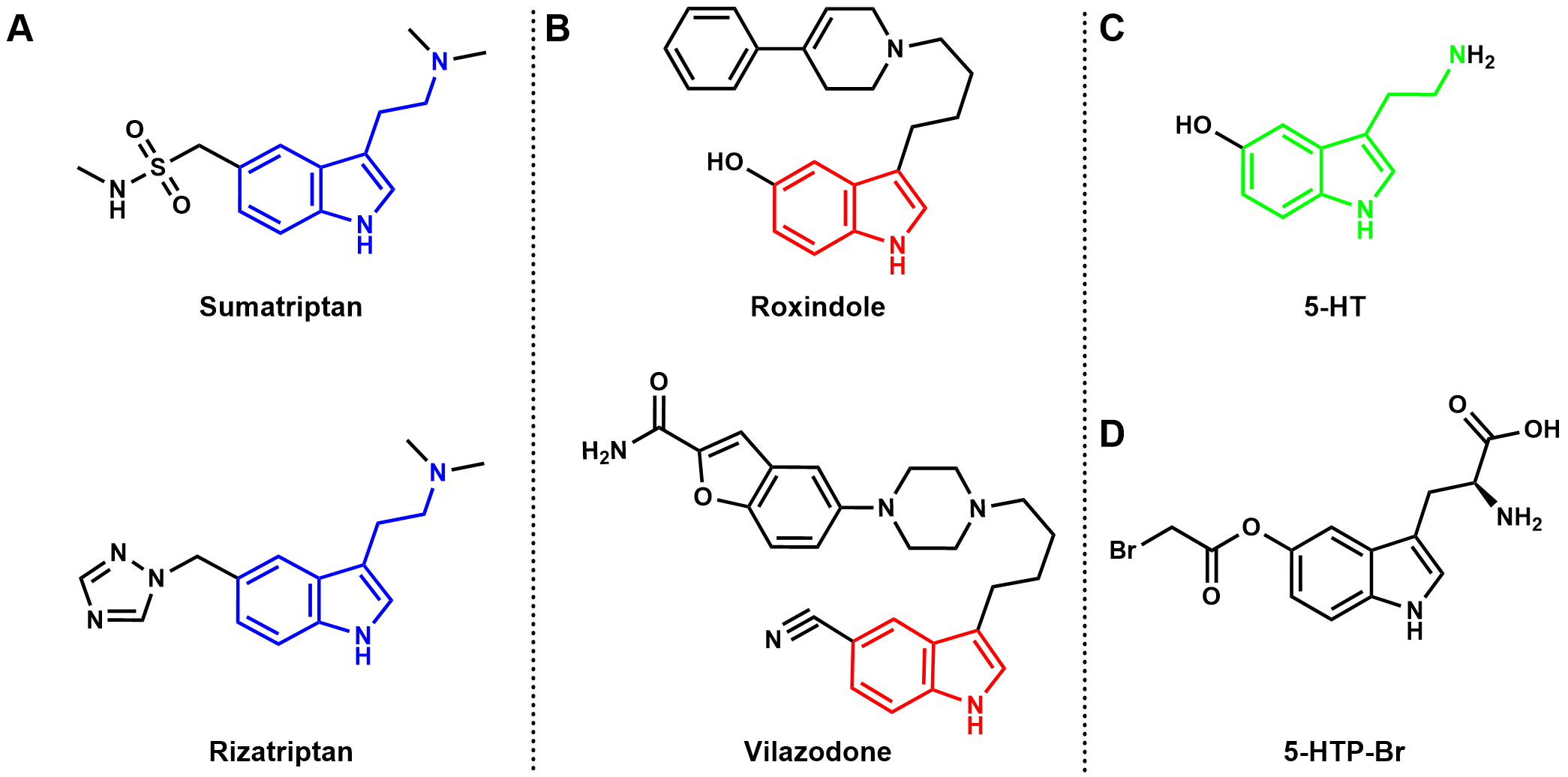


**Figure S1. Structural comparison highlights pharmacophores for dual targeting of SERT and 5-HTRs.** **(A).** Representative synthetic 5-HTR ligands (Sumatriptan, Rizatriptan) share a conserved indoleamine moiety (highlighted in blue) for receptor recognition and binding. **(B).** Representative synthetic compounds (Roxindole, Vilazodone) for targeting both 5-HTRs and SERT exhibit a conserved indole core (highlighted in red), suggesting a shared pharmacophore that supports engagement of both targets. **(C).** The endogenous neurotransmitter 5-HT is an agonist of 5-HTRs and a substrate of SERT. **(D).** The 5-HTP is functionalized with a carbonyl bromide group to generate a carbonyl bromide-modified derivative (5-HTP-Br), providing a conjugation handle for SeroPTEN-CG construction while preserving the indoleamine pharmacophore and AADC-mediated conversion to 5-HT. **Abbreviations:** 5-HT, 5-hydroxytryptamine; 5-HTP, 5-hydroxytryptophan; 5-HTR, 5-hydroxytryptamine receptor; SERT, serotonin transporter; AADC, aromatic L-amino acid decarboxylase.


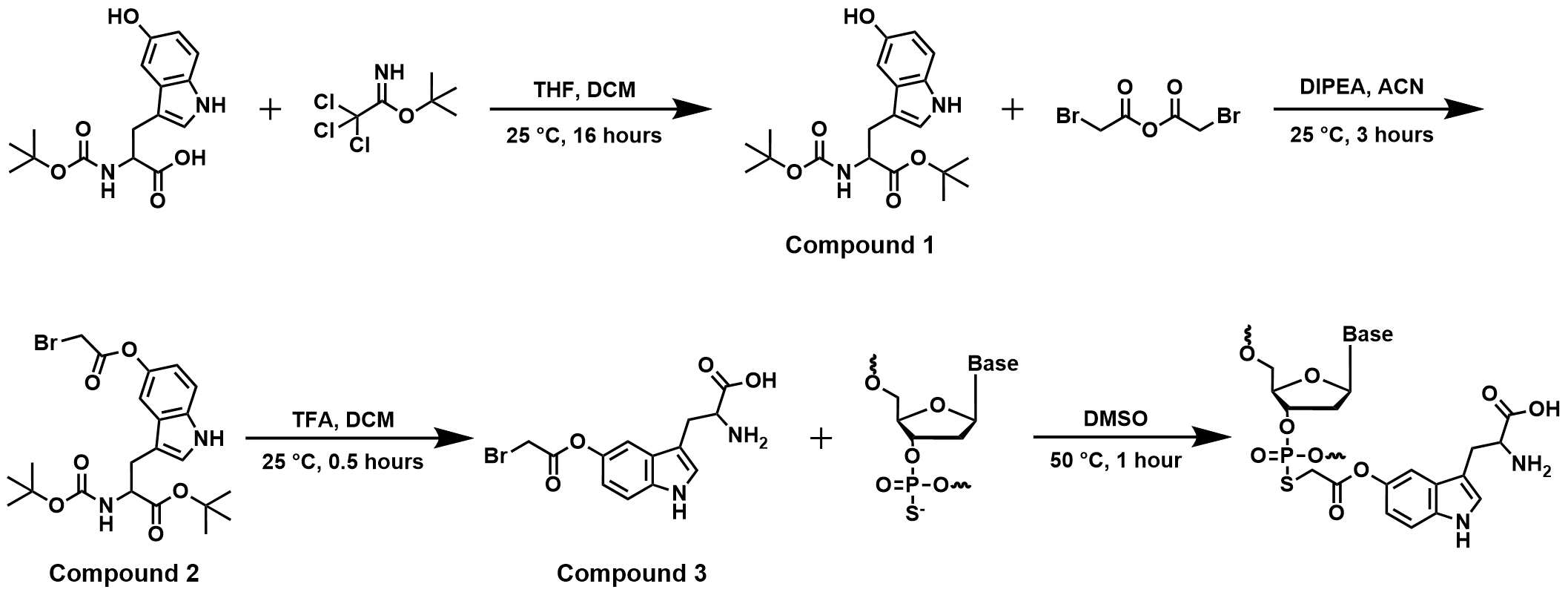


**Figure S2. The synthetic routes of 5-HTP-Br (Compound 3) and 5-HTP-DNA conjugates. Abbreviations:** THF, tetrahydrofuran; DCM, dichloromethane; DIPEA, N,N-diisopropylethylamine; TFA, trifluoroacetic acid; DMSO, dimethyl sulfoxide.


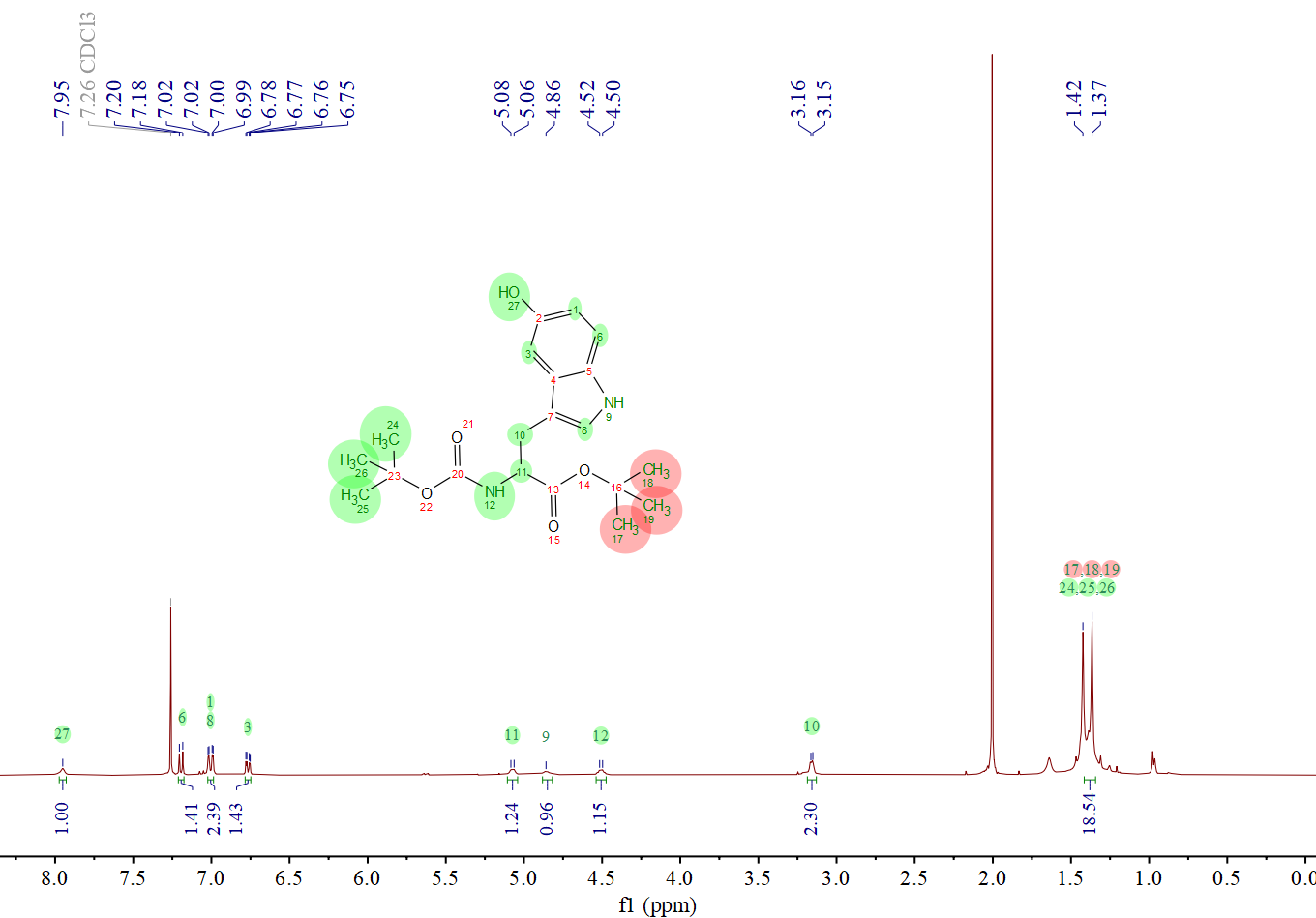


**Figure S3. ^1^H NMR spectra of Compound 1.**


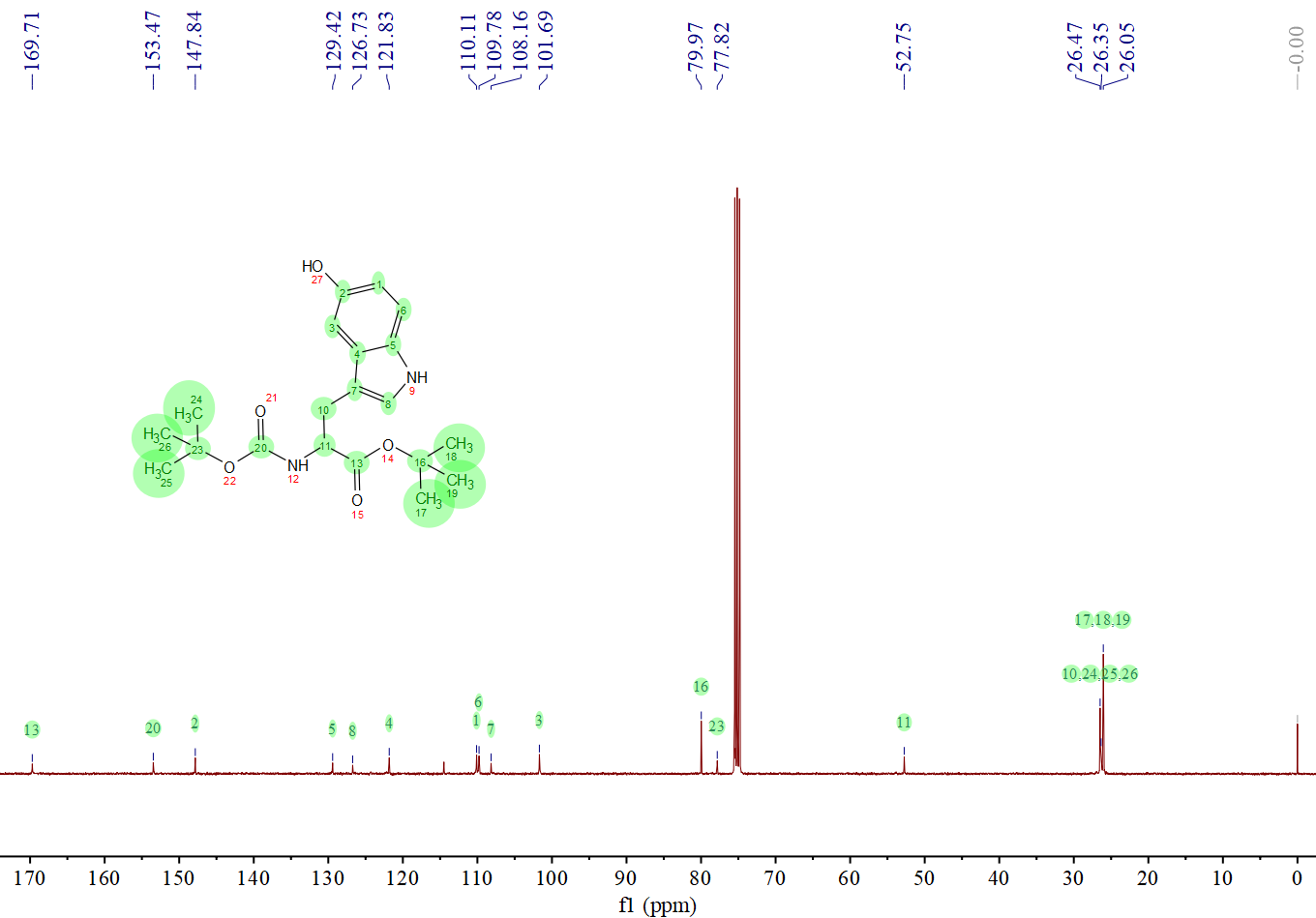


**Figure S4. ^13^C NMR spectra of Compound 1.**


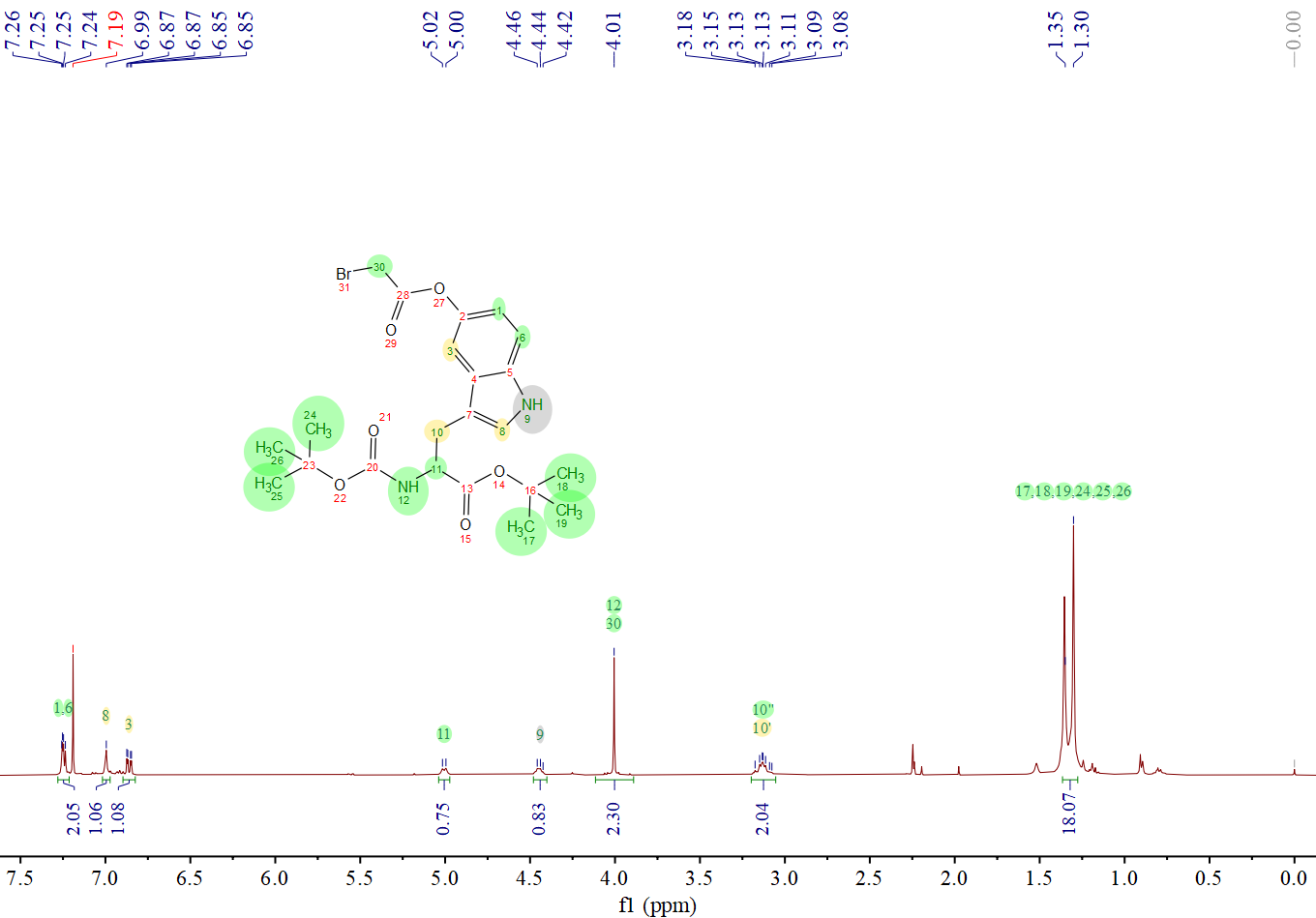


**Figure S5. ^1^H NMR spectra of Compound 2.**


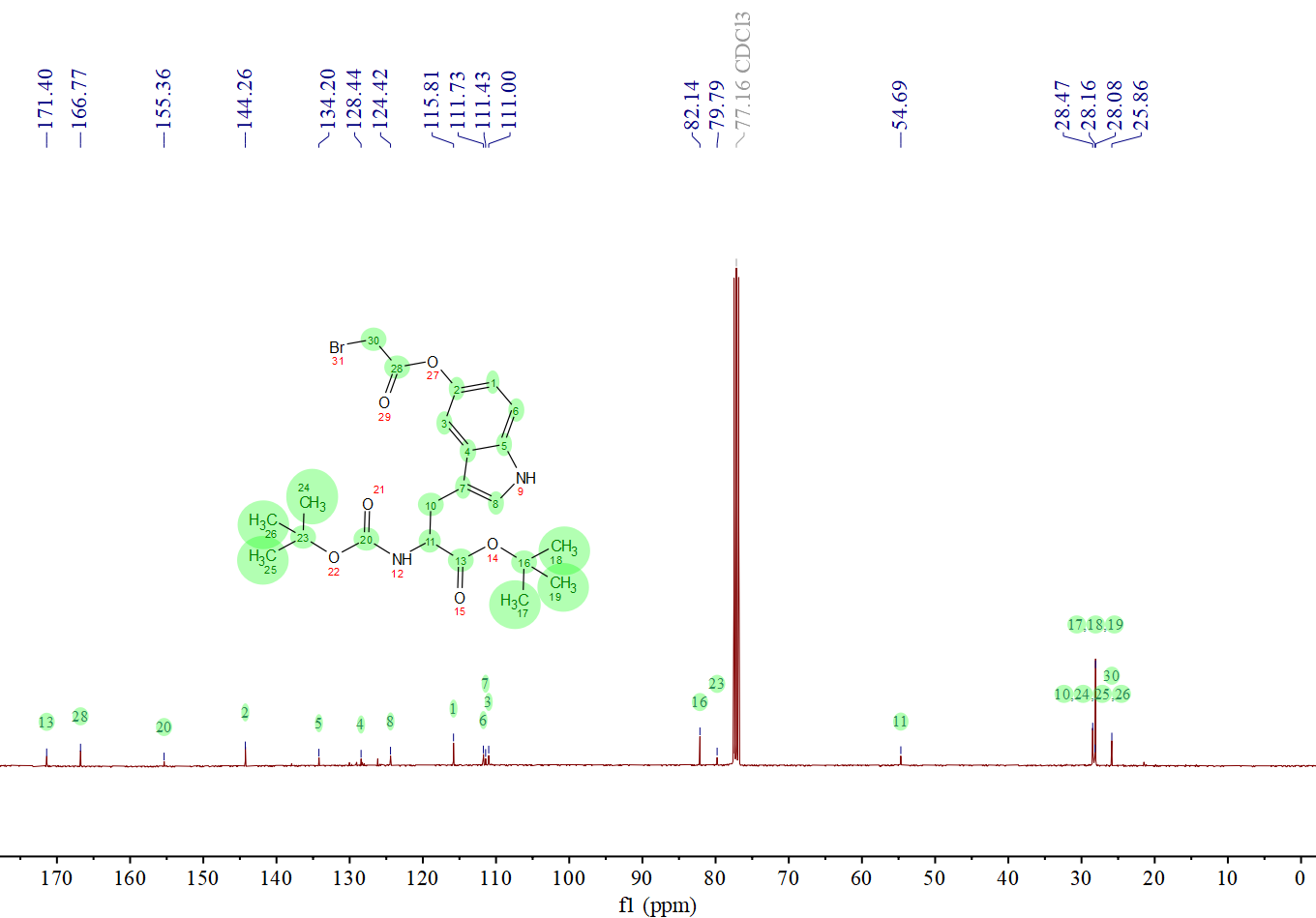


**Figure S6. ^13^C NMR spectra of Compound 2.**

**
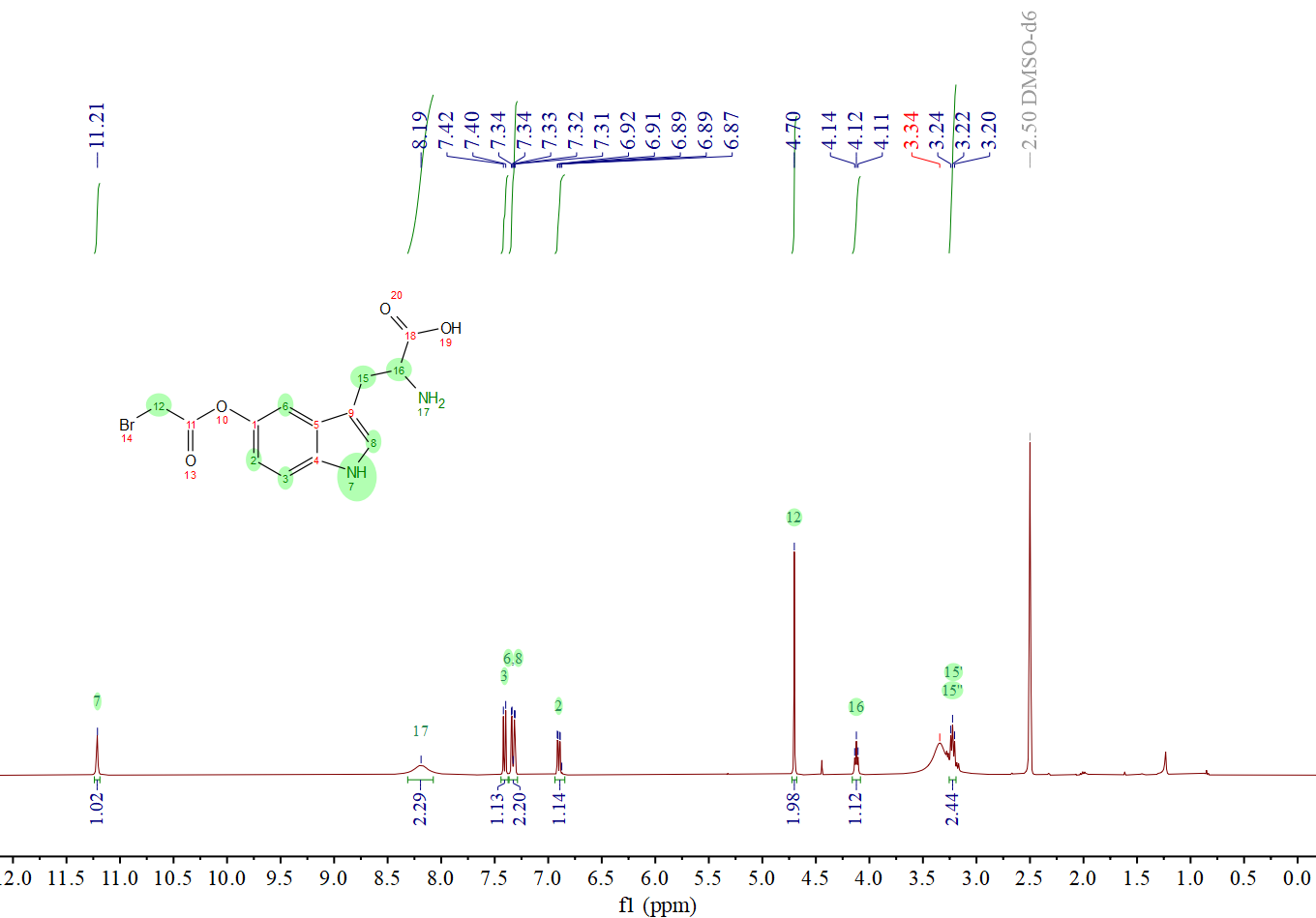
**

**Figure S7. ^1^H NMR spectra of Compound 3.**


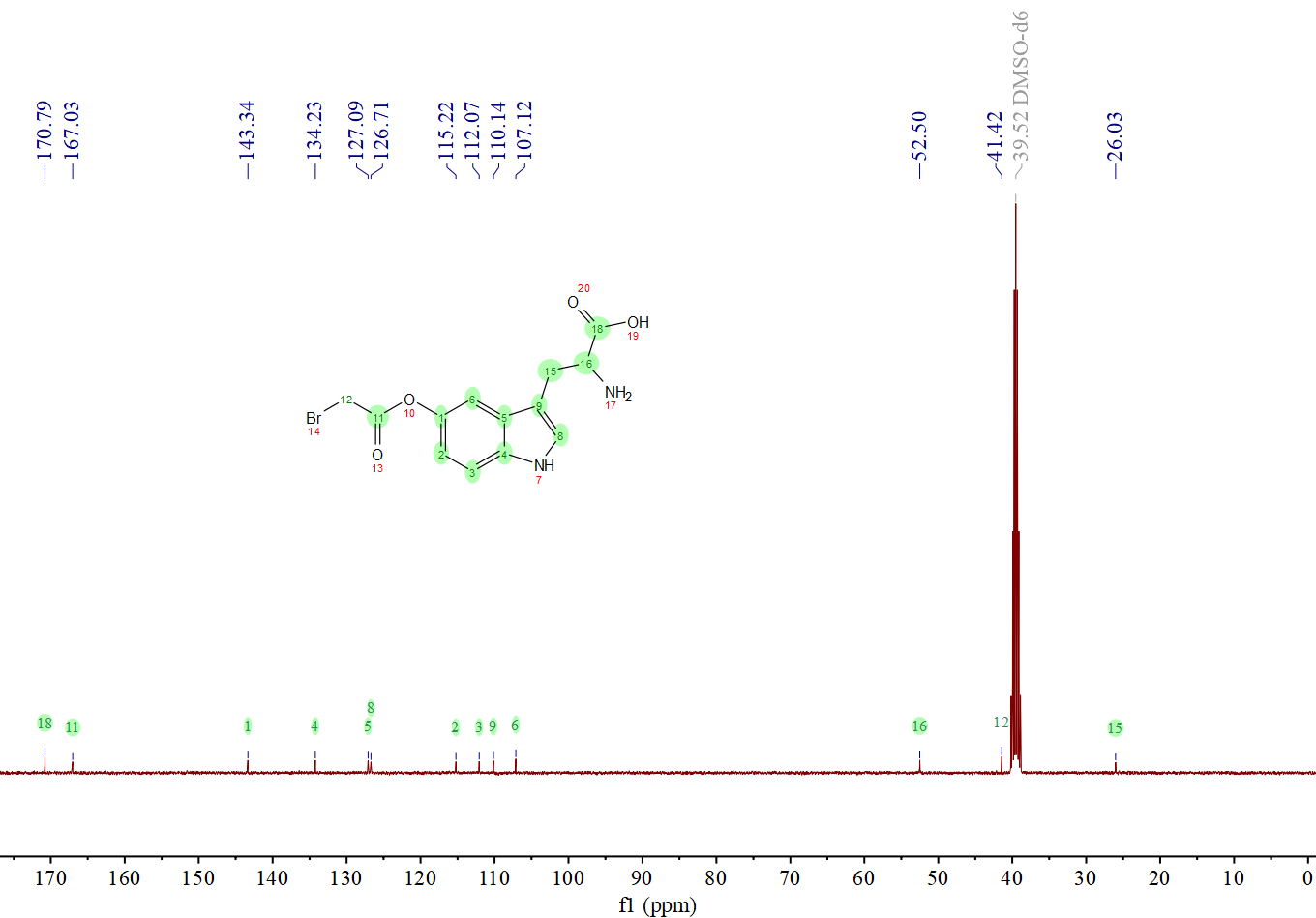


**Figure S8. ^13^C NMR spectra of Compound 3.**


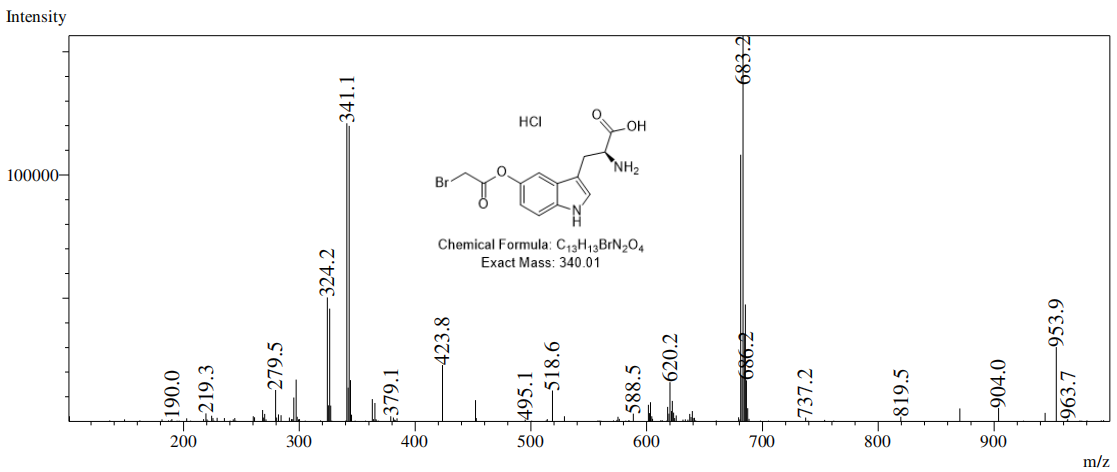


**Figure S9. The MS spectra of Compound 3.**


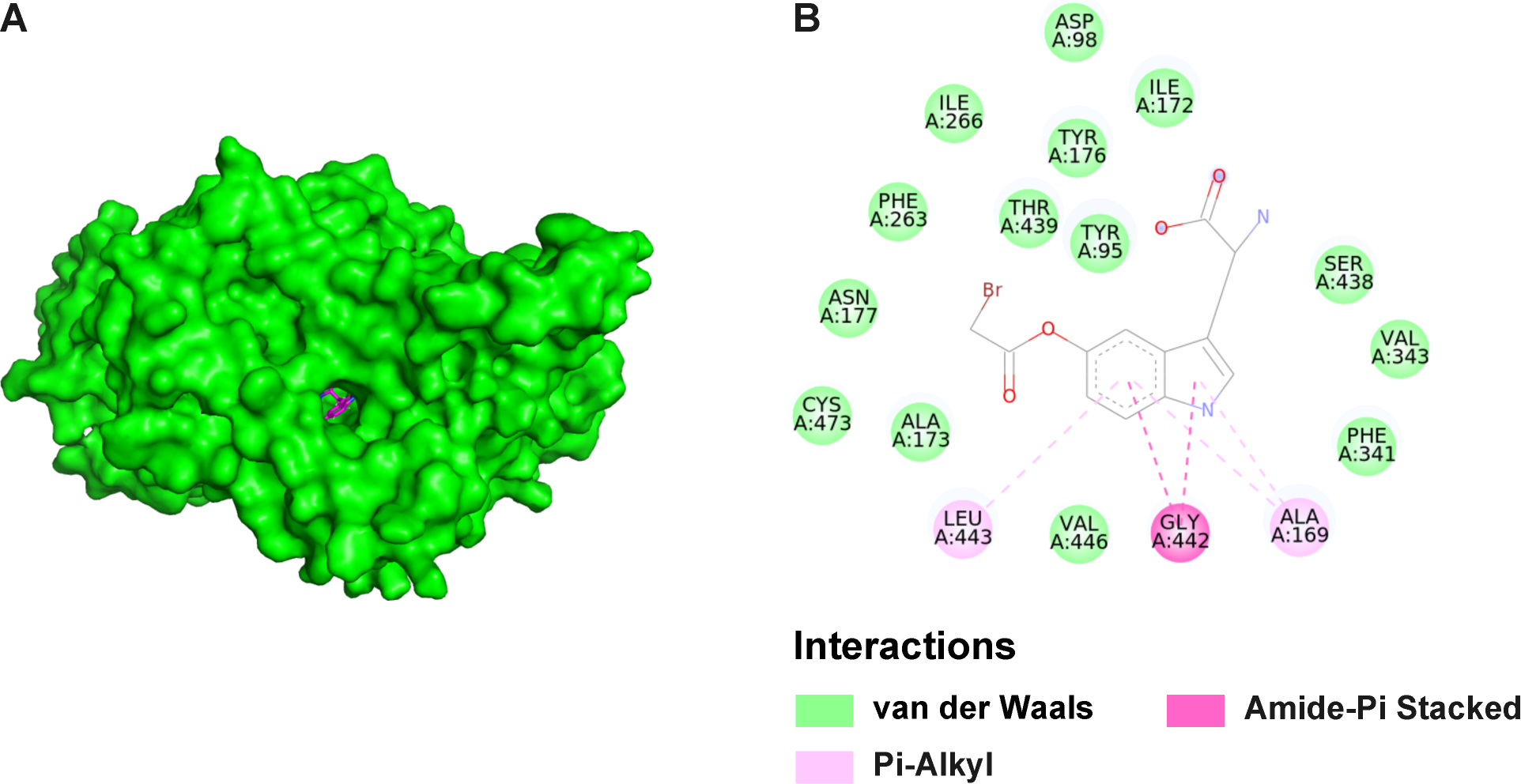


**Figure S10. The docking complex of 5-HTP-Br and SERT. (A).** The predicted conformation of the complex. **(B).** The 2D detailed view shows the interactions between 5-HTP-Br and neighboring residues of SERT.


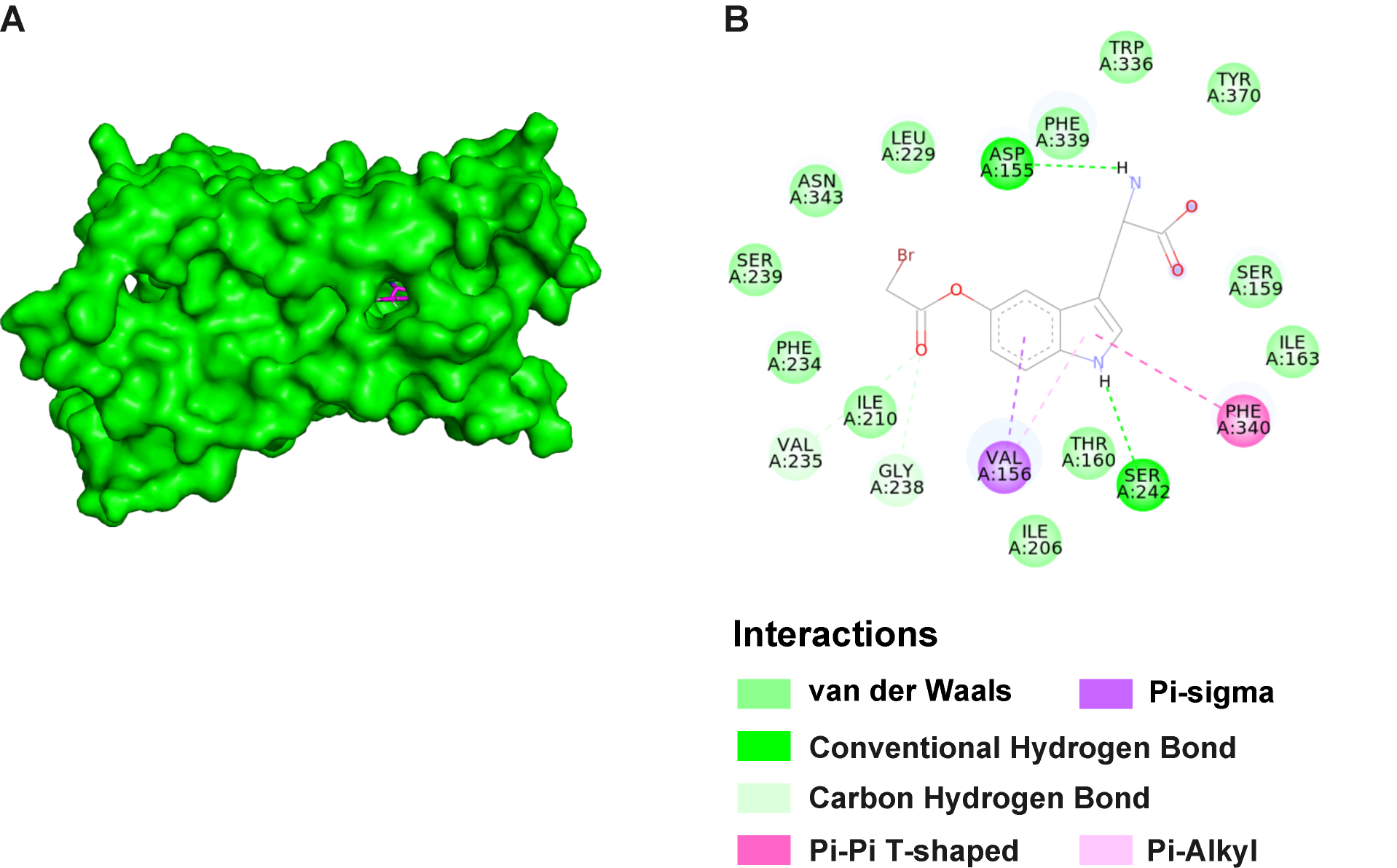


**Figure** **S11. The docking complex of 5-HTP-Br and 5‑HT_2A_R. (A).** The predicted conformation of the complex. **(B).** The 2D detailed view shows the interactions between 5-HTP-Br and neighboring residues of 5‑HT_2A_R.


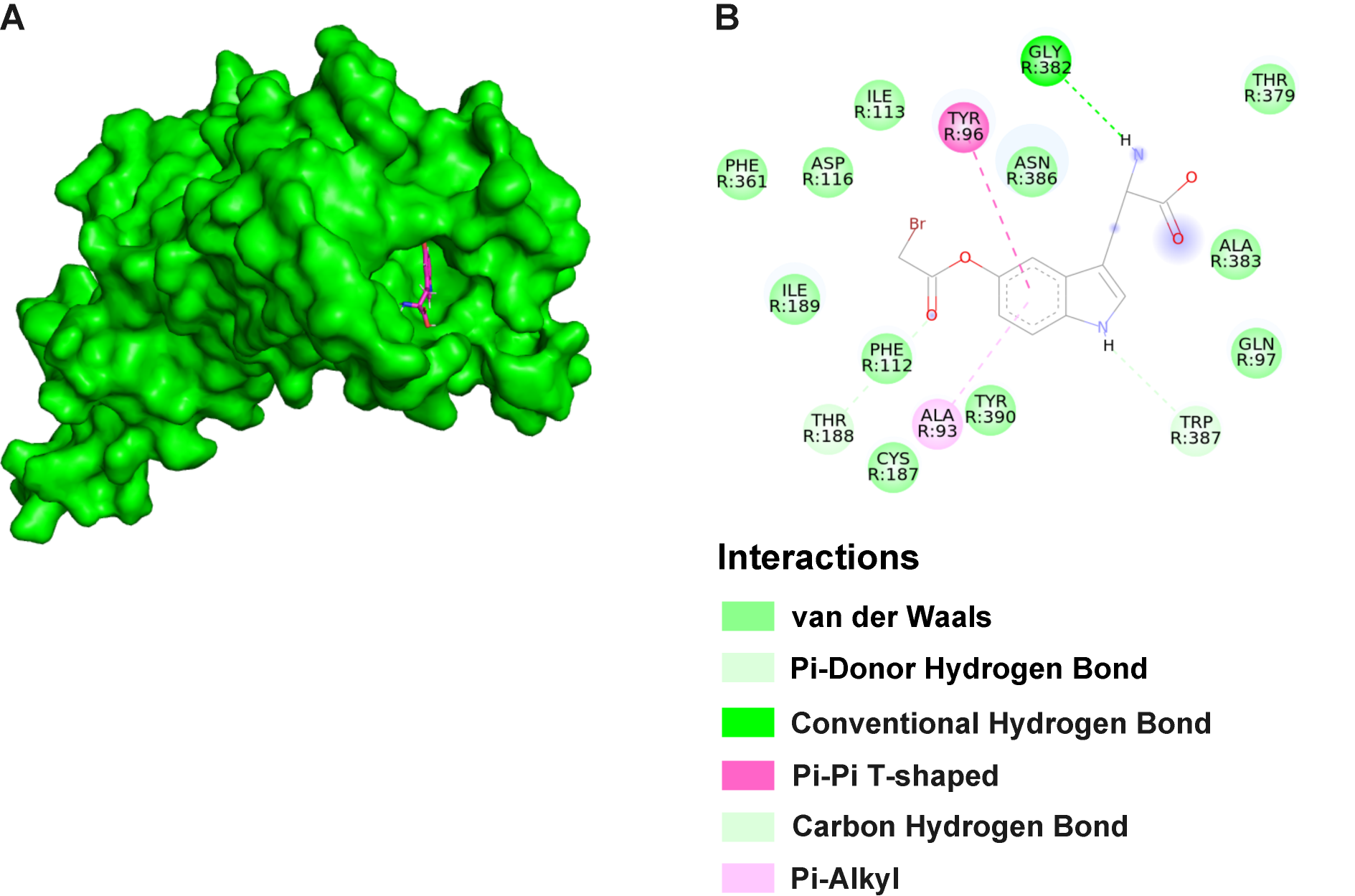


**Figure S12. The docking complex of 5-HTP-Br and 5‑HT_1A_R. (A).** The predicted conformation of the complex. **(B).** The 2D detailed view shows the interactions between 5-HTP-Br and neighboring residues of 5‑HT_1A_R.


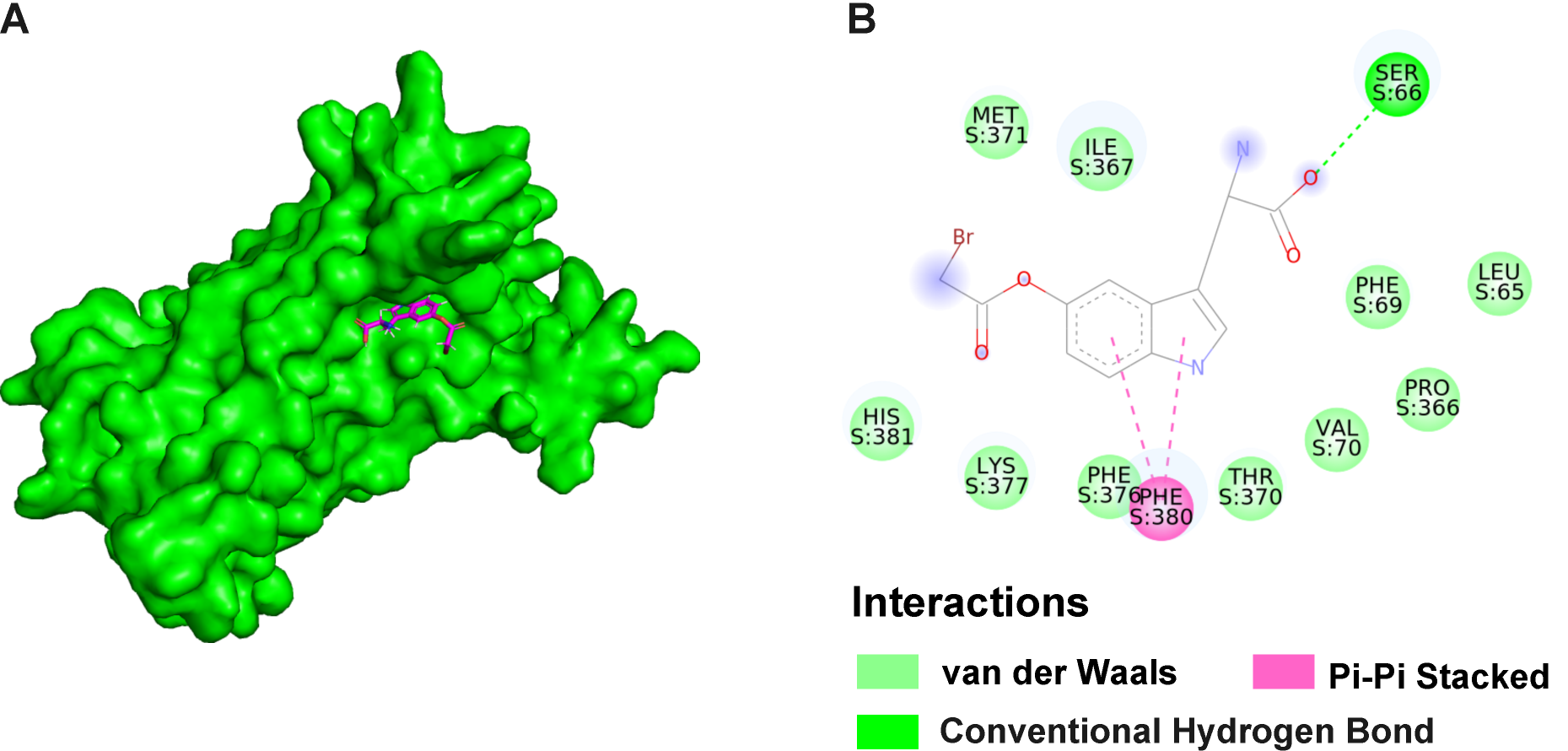


**Figure S13. The docking complex of 5-HTP-Br and 5‑HT_1B_R. (A).** The predicted conformation of the complex. **(B).** The 2D detailed view shows the interactions between 5-HTP-Br and neighboring residues of 5‑HT_1B_R.


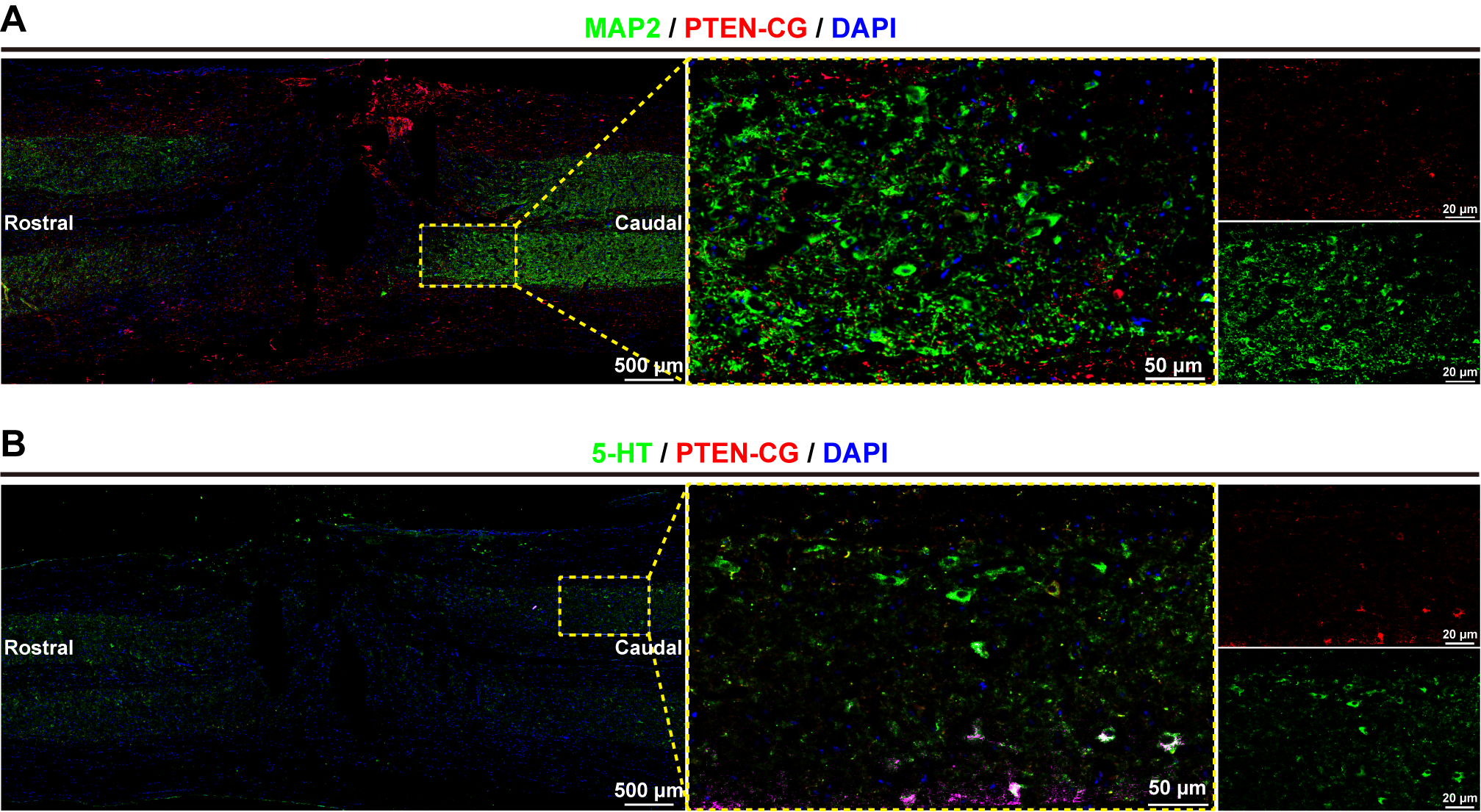


**Figure S14.** **Lack of neuronal and serotonergic system targeting by PTEN-CG in vivo. (A).** Representative confocal images of the spinal cord lesion site along the rostral-caudal axis showing MAP2 (green) and Cy5.5-PTEN-CG (red). The zoomed-in view (middle panels) and corresponding single-channel images (right) reveal little colocalization of PTEN-CG with MAP2⁺ neurons. **(B).** Parallel staining for 5-HT (green) and Cy5.5-PTEN-CG (red) reveals little colocalization of PTEN-CG with serotonergic neurons at the lesion site. Scale bars, as indicated. **Abbreviations:** MAP2, microtubule-associated protein 2; 5-HT, 5-hydroxytryptamine.


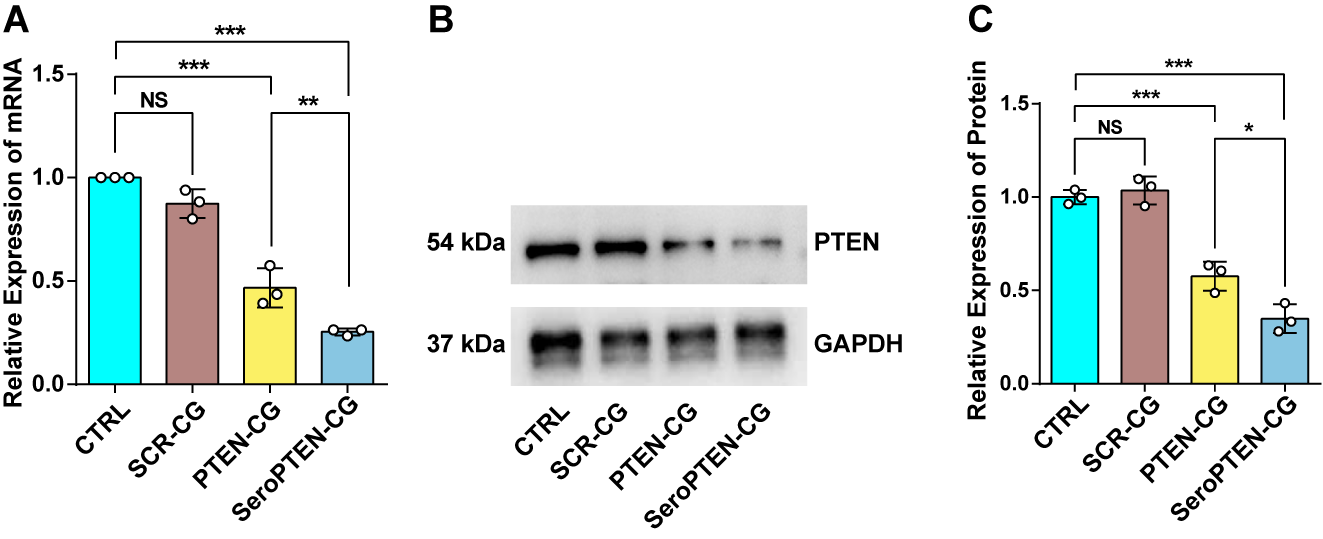


**Figure S15. SeroPTEN-CG achieves PTEN silencing in neurons. (A).** qRT-PCR quantification of *Pten* mRNA in primary neurons after treatment with PBS, SCR-CG, PTEN-CG, or SeroPTEN-CG, expression values are normalized to GAPDH and presented relative to CTRL (n = 3 per group). **(B, C).** PTEN silencing in primary neurons after incubation with the indicated formulations (SCR-CG, PTEN-CG, and SeroPTEN-CG), with a representative western blot (B) and densitometric quantification normalized to GAPDH (C) (n = 3 per group). Quantitative data in (A, C) are presented as mean ± SEM. Statistical significance is assessed using one-way ANOVA with Tukey’s post hoc test for multiple comparisons. ^*^*p* < 0.05, ^**^*p* < 0.01, ^***^*p* < 0.001; NS, not significant.


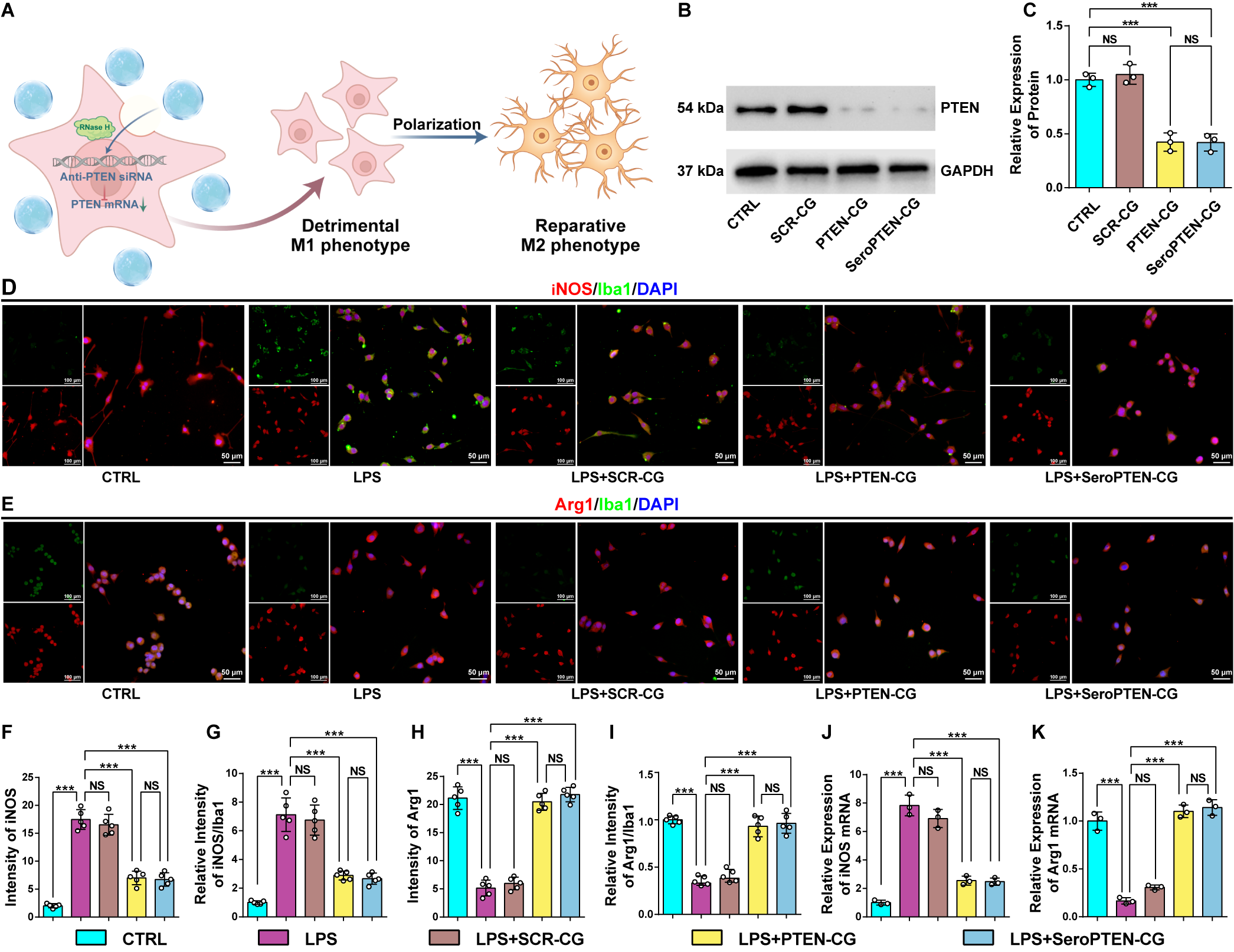


**Figure S16. SeroPTEN-CG reprograms microglia toward a reparative M2 phenotype. (A).** The scheme illustrates that off-target uptake of SeroPTEN-CG nanogels by activated M1 microglia drives polarization toward a reparative M2 phenotype. **(B, C).** PTEN silencing in microglia after incubation with the indicated formulations (SCR-CG, PTEN-CG, and SeroPTEN-CG), with a representative western blot (B) and densitometric quantification normalized to GAPDH (C), (n = 3 per group). **(D-E).** Immunofluorescence images of LPS-primed microglia stained for iNOS/Iba1/DAPI (D) and Arg1/Iba1/DAPI (E) across the indicated treatment groups. **(F-I).** Quantifications show that SeroPTEN-CG significantly decreases iNOS intensity (F) and the iNOS/Iba1 index (G) while increasing Arg1 intensity (H) and the Arg1/Iba1 index (I), (n = 5 per group). **(J, K).** qRT-PCR analysis of iNOS (J) and Arg1 (K) mRNA confirms suppression of iNOS and induction of Arg1 in PTEN-CG and SeroPTEN-CG groups (n = 3 per group). Scale bars, as indicated. Quantitative data in (C, F-K) are presented as mean ± SEM. Statistical significance is assessed using one-way ANOVA with Tukey’s post hoc test for multiple comparisons. ^*^*p* < 0.05, ^**^*p* < 0.01, ^***^*p* < 0.001; NS, not significant. **Abbreviations:** iNOS, inducible nitric oxide synthase; Iba1, ionized calcium binding adapter molecule 1; Arg1, arginase-1; LPS, lipopolysaccharide.


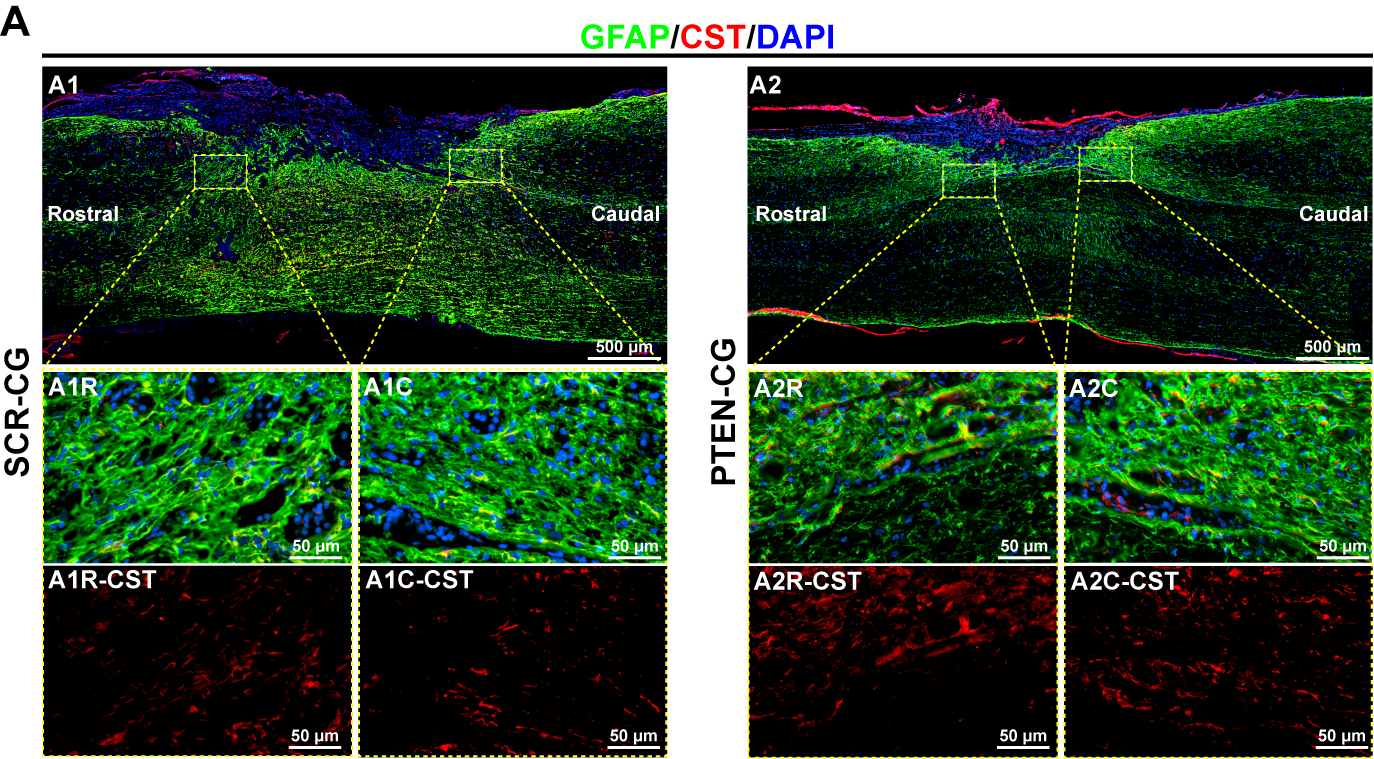


**Figure S17. CST architecture at rostral and caudal lesion borders after SCI.** Representative spinal sections from SCR-CG and PTEN-CG groups show the astroglial network (GFAP, green) and CST (AAV9-hSyn-mCherry, red). Insets (A1R, A1C, A2R, and A2C) display rostral and caudal ROIs, and the corresponding single-channel CST images are shown below (A1R-CST, A1C-CST, A2R-CST, and A2C-CST). Scale bars, as indicated. **Abbreviations:** GFAP, glial fibrillary acidic protein; CST, corticospinal tract; ROI, region of interest.


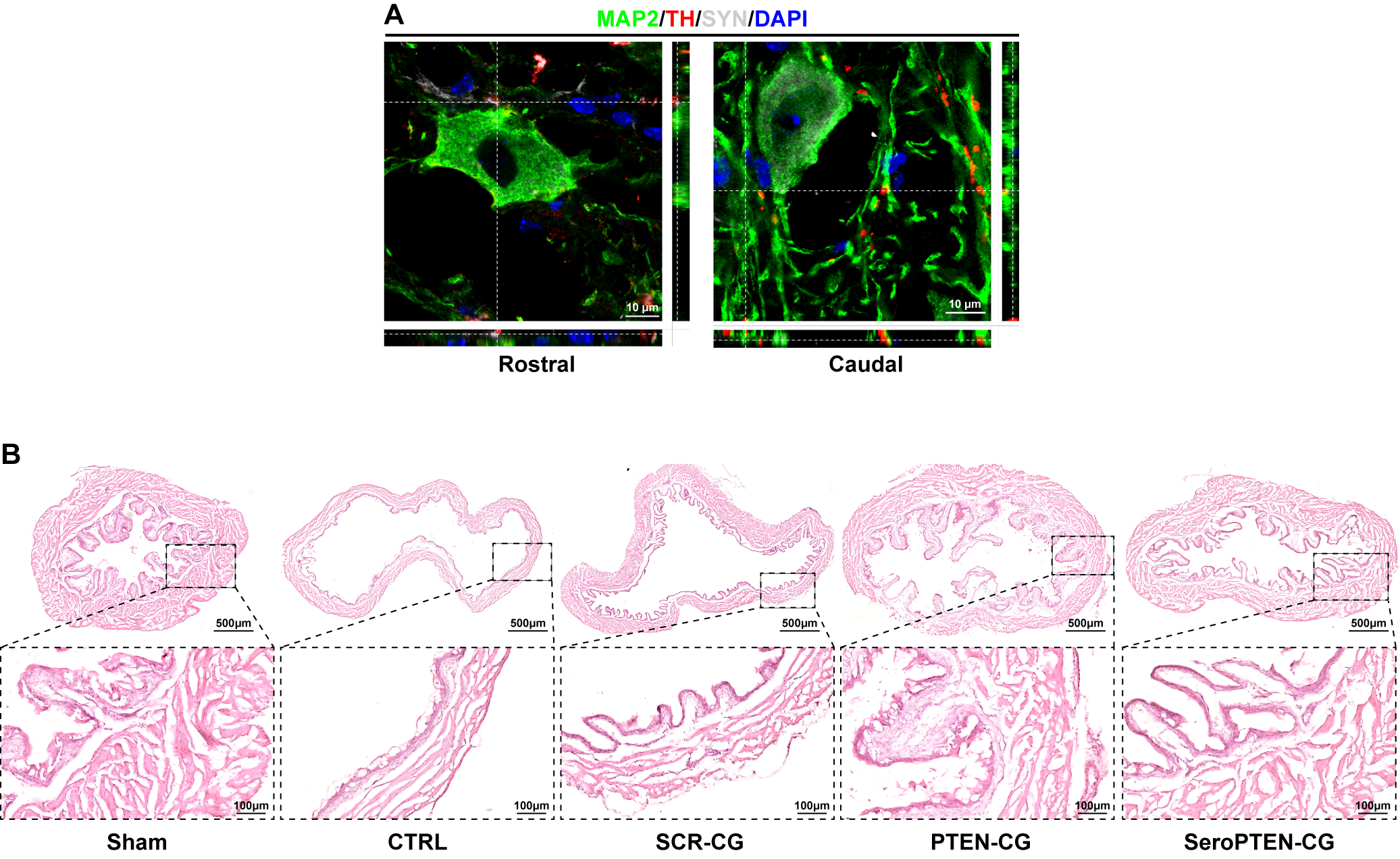


**Figure S18.** **Synaptic reestablishment at lesion sites and improvement of bladder pathology after SeroPTEN-CG treatment. (A).** High-magnification images with TH (red), MAP2⁺ neurons (MAP2, green), synaptophysin (SYN, gray) at rostral and caudal lesion borders in the SeroPTEN-CG group. Orthogonal views (right and bottom strips) highlight colocalization of TH, MAP2, and SYN, indicating the reestablished inputs after SeroPTEN-CG treatment. **(B).** H&E staining of whole-mount urinary bladders from Sham, CTRL, SCR-CG, PTEN-CG, and SeroPTEN-CG groups with high-magnification insets (bottom). Scale bars, as indicated. **Abbreviations:** TH, tyrosine hydroxylase; MAP2, microtubule-associated protein 2; SYN, synaptophysin; H&E, hematoxylin and eosin.


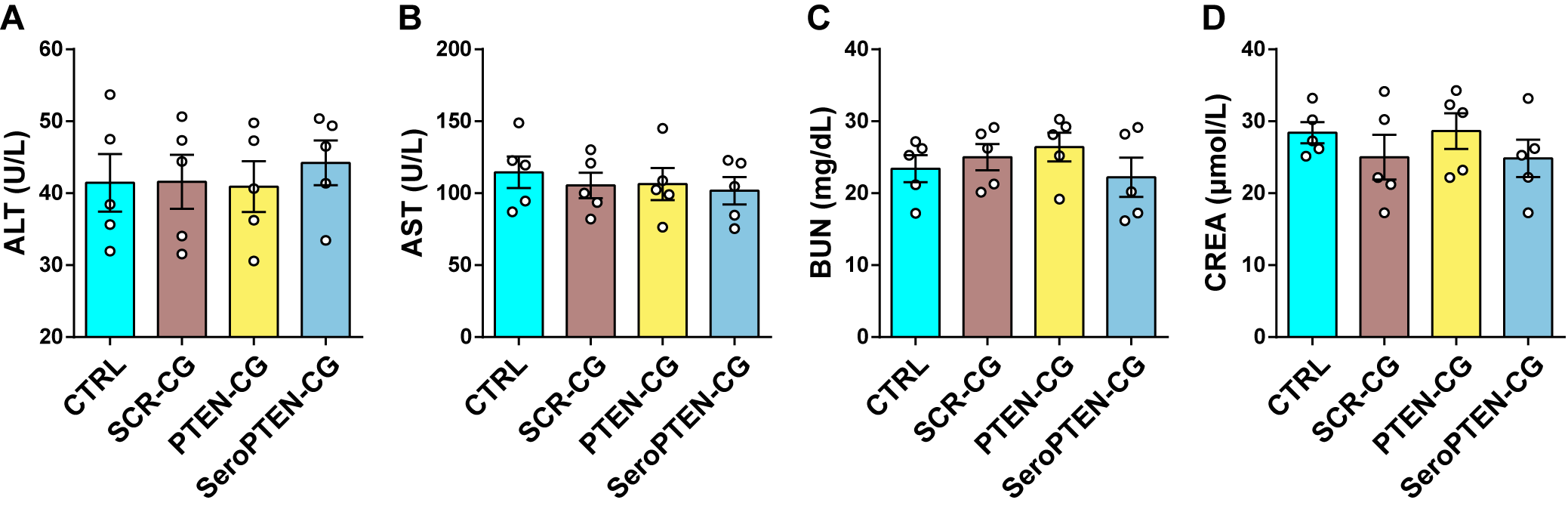


**Figure S19. Serum biochemistry indicates no detectable hepatotoxicity or nephrotoxicity after treatment.** **(A-D).** Serum ALT (A), AST (B), BUN (C), and CREA (D) were measured at the study endpoint in CTRL, SCR-CG, PTEN-CG, and SeroPTEN-CG groups (n = 5 per group). Quantitative data are presented as mean ± SEM. Statistical significance was assessed using one-way ANOVA with Tukey’s post hoc test for multiple comparisons. No significant differences were detected among groups (*p* > 0.05), therefore no significance symbols are shown. **Abbreviations:** ALT, alanine aminotransferase; AST, aspartate aminotransferase; BUN, blood urea nitrogen; CREA, creatinine.


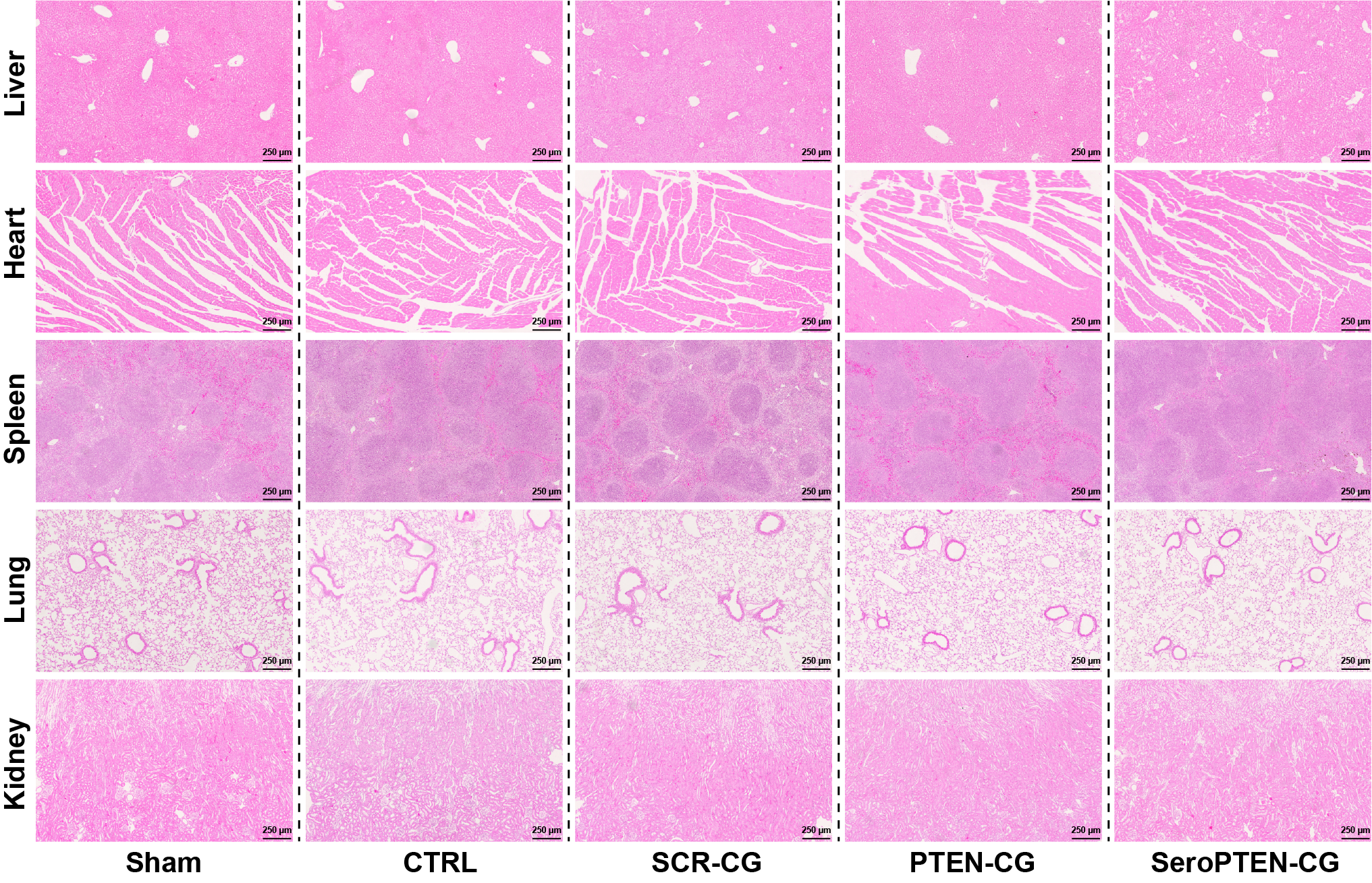


**Figure S20. Histopathological evaluation indicates no detectable toxicity in major organs after treatment.** Representative H&E-stained sections of liver, heart, spleen, lung, and kidney from Sham, CTRL, SCR-CG, PTEN-CG, and SeroPTEN-CG groups (n = 5 per group). Across all groups, tissue architecture is preserved, with no necrosis or diffuse inflammatory cell infiltration. Scale bars, as indicated. **Abbreviations:** H&E, hematoxylin and eosin.

**Supplementary Tables**

**Table S1.** **Oligonucleotide sequences used in this study**

| Oligonucleotides | Oligonucleotide sequences (5’- 3’) |
| --- | --- |
| Ya-2PS | TGGATCC*GCATGACATTCGCC*GTAAGTTGACCTGTGAA |
| Yb-2PS | CTTACGG*CGAATGACCGAATC*AGCCTTTGACCTGTGAA |
| Yc-2PS | AGGCTGA*TTCGGTTCATGCGG*ATCCATTGACCTGTGAA |
| Sense strand  (scramble siRNA) | rCrGrGrUrGrArGrCrCrArGrGrCrGrUrGrCrArArUrUrUrUrCrArCrArGrGrUrCrArA |
| Antisense strand  (scramble siRNA) | rArArUrUrGrCrArCrGrCrCrUrGrGrCrUrCrArCrCrGrUrUrCrArCrArGrGrUrCrArA |
| Sense strand  (PTEN siRNA) | rGrGrGrUrUrUrGrArUrArArGrUrUrCrUrArGrCrUrUrCrArCrArGrGrUrCrArA |
| Antisense strand (PTEN siRNA) | rGrCrUrArGrArArCrUrUrArUrCrArArArCrCrCrUrUrCrArCrArGrGrUrCrArA |
| Forward primer  (PTEN) | GGGTTTGATAAGTTCTAGC |
| Reverse primer  (PTEN) | GCTAGAACTTATCAAACCC |
| Forward primer  (GAPDH) | CAGGAGGCATTGCTGATGAT |
| Reverse primer  (GAPDH) | GAAGGCTGGGGCTCATTT |

a) * represents the site with phosphorothioate modifications.

b) Underlined sequences indicate sticky ends designed for hybridization.

c) In cellular uptake study, the FAM and Cy5.5 fluorophores are labeled at the 3’ terminus on sense strand of siRNA.
